# Vancomycin eliminates gut deoxycholic acid, restoring ER proteostasis in ILC2s and relieving colitis

**DOI:** 10.1172/jci.insight.197470

**Published:** 2026-04-08

**Authors:** Qiuheng Tian, Han Liu, Xiang Gu, Jing Shen, Xi Yuan, Mengqi Zheng, Yunjiao Zhai, Yatai Chen, Penghu Han, Yangchun Ma, Wei Xin, Hongyue Ma, Yu Li, Sihan Wang, Lei Guo, Detian Yuan, Yanbo Yu, Shiyang Li

**Affiliations:** 1 Department of Gastroenterology, Qilu Hospital of Shandong University, Advanced Medical Research Institute, Shandong University, Jinan, China.; 2 Department of Gastroenterology, Qilu Hospital (Qingdao), Cheeloo College of Medicine, Shandong University, Qingdao, Shandong, China.; 3 Shandong Provincial Clinical Research Center for Digestive Diseases, Jinan, China.; 4 Shandong University Suzhou Research Institute and; 5 Department of Medicinal Chemistry, Key Laboratory of Chemical Biology (Ministry of Education), Cheeloo College of Medicine, Shandong University, Jinan, China.; 6 Children’s Hospital Affiliated to Shandong University (Jinan Children’s Hospital), Jinan, China.; 7 Department of Biochemistry and Molecular Biology, School of Basic Medical Sciences, and; 8 Key Laboratory for Experimental Teratology of Ministry of Education, Shandong University, Jinan, China.

**Keywords:** Gastroenterology, Immunology, Inflammatory bowel disease, Innate immunity, Protein misfolding

## Abstract

Ulcerative colitis (UC) remission is marked by gut microbiota restructuring, but how microbial metabolites influence immune-mediated tissue repair is unclear. Here, we demonstrate that oral vancomycin alleviates colitis symptoms in murine models, mirroring its clinical efficacy in inducing remission in patients with UC. Mechanistically, vancomycin’s therapeutic effect is achieved by reducing deoxycholic acid (DCA). We reveal that DCA impairs mucosal repair driven by group 2 innate lymphoid cells (ILC2s) by inducing ER stress through direct binding to thioredoxin-related transmembrane protein 2 (TMX2). This interaction disrupts TMX2’s role in protein folding, triggering unresolved unfolded protein response via hyperactivation of PERK/eIF2α signaling, which suppresses the production of pro-healing molecules by ILC2s. Pharmacological inhibition of PERK phosphorylation restores ILC2 function and accelerates colitis resolution. Our work uncovers a pathogenic microbiota/DCA/ILC2 axis that obstructs mucosal healing and positions vancomycin as a targeted strategy to eliminate DCA, thereby promoting UC remission.

## Abstract 2

Gut commensals-derived DCA compromises ILC2-mediated tissue repair via disrupting endoplasmic reticulum proteostasis.

## Introduction

Microbial ecosystem reconfiguration is a hallmark of ulcerative colitis (UC) remission (1), yet how specific microbiota-derived metabolites influence tissue-repairing immune circuits remains poorly understood. This knowledge gap contrasts with intriguing clinical observations that oral vancomycin, an antibiotic targeting Gram-positive bacteria, induces sustained remission in UC (2–7), despite the general contraindication of antibiotics in colitis unless severe bacterial infection occurs. This therapeutic effect may be linked to vancomycin’s capacity to eliminate pathobionts and remodel bile acid metabolism, particularly depleting the secondary bile acid (8). Given its negligible systemic absorption, the therapeutic benefits of oral vancomycin likely stem from its impact on the gut microbiome, warranting further investigation.

The gut tissue repair process orchestrates complex cellular choreography involving multiple immune elements (9–11), with group 2 innate lymphoid cells (ILC2s) emerging as pivotal regulators through secreting epithelium-repairing mediators including amphiregulin (Areg), IL-13, and IL-5 (12–14). Although transcriptional regulation of ILC2s has been extensively characterized (15–17), their dynamic crosstalk with the tissue microenvironment during UC remission remains elusive.

Here, we identify deoxycholic acid (DCA) elimination as the central mechanism underlying vancomycin’s therapeutic efficacy. Our results demonstrate that microbiota-derived DCA impedes colitis remission by subverting ILC2 functionality through a conventional receptor-independent pathway. Mechanistically, DCA directly interacts with ER-resident thioredoxin-related transmembrane protein 2 (TMX2), thereby provoking unresolved unfolded protein response (UPR). Furthermore, activation of the PERK/eIF2α arm of UPR compromises ILC2-mediated mucosal repair. Therapeutically, pharmacological inhibition of PERK phosphorylation restores ILC2 function and accelerates colitis recovery in DCA-challenged models. Our work not only deciphers a microbiota/ILC2 metabolic axis governing mucosal healing, but also positions vancomycin as a precision modulator of pathogenic microbial metabolites for UC treatment.

## Results

### Vancomycin promotes colitis remission by eliminating DCA.

**Figure 1:**
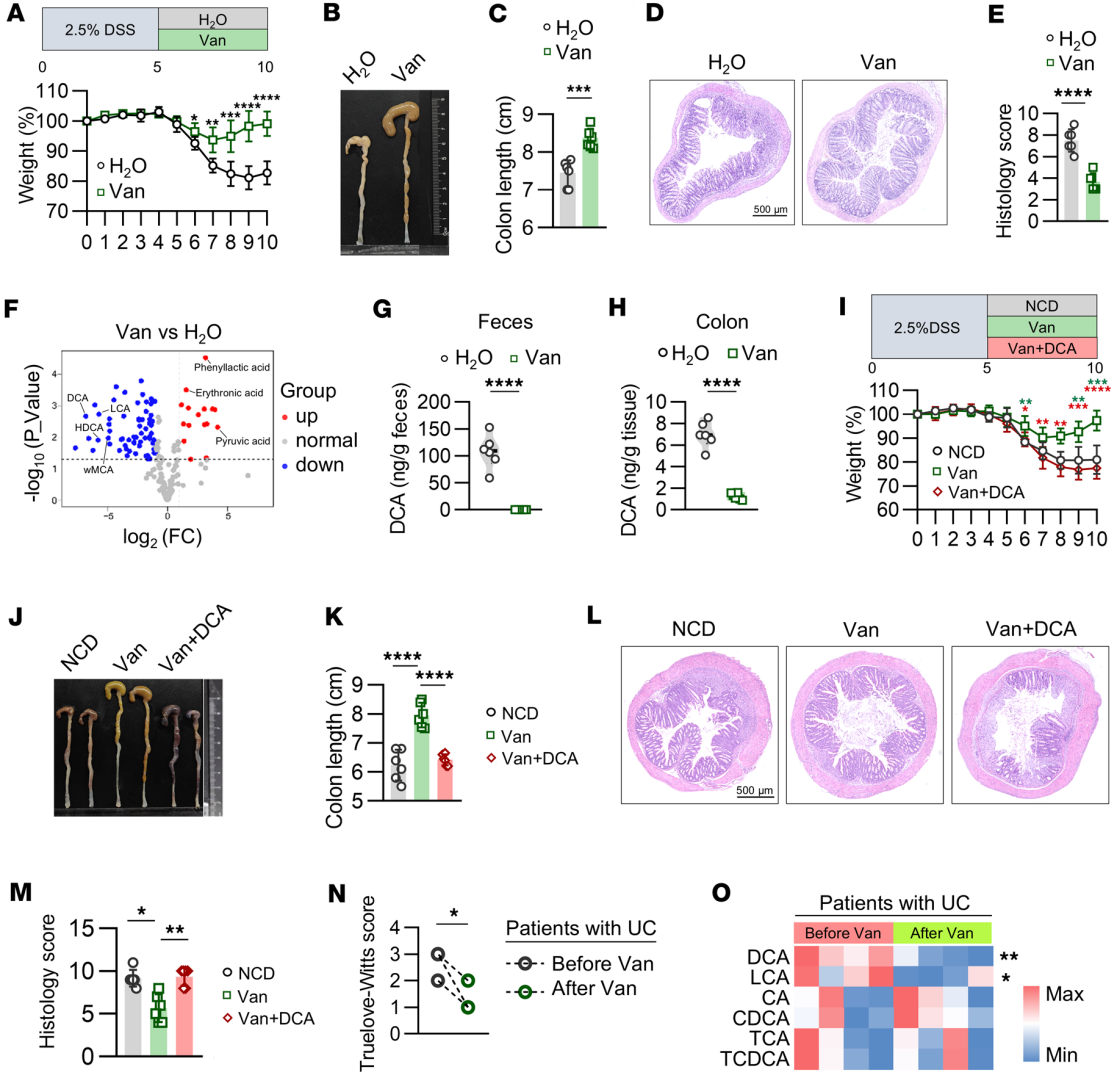
Vancomycin promotes colitis remission by eliminating DCA. (**A**–**E**) WT mice received 5 days of H_2_O or vancomycin by gavage after 5 days of 5% DSS. (**A**, top) Experimental outline. (**A**, bottom) Weight course. (**B**) Representative image of colon. (**C**) Statistical data of colon length. (**D**) H&E-stained histological sections. Scale bar: 500 μm. (**E**) Histopathology scores. *n* = 6 mice per group; experiment repeated 3 times. (**F**) Metabolite profiles in vancomycin-treated compared with H_2_O-treated mice. (**G**) DCA in feces. (**H**) Colon tissue after vancomycin gavage. *n* = 6 mice per group; experiment repeated 3 times. (**I**–**M**) WT mice received 5 days of H_2_O or vancomycin after 5 days of 2.5% DSS. One group of vancomycin-treated mice was simultaneously fed 2% DCA diet on days 5 to 10. (**I**, top) Experimental outline. (**I**, bottom) Weight course. Green asterisk: comparison between NCD and Van groups; red asterisk: comparison between Van and Van+DCA groups. (**J**) Representative image of colon. (**K**) Statistical data of colon length. (**L**) H&E-stained histological sections. Scale bar: 500 μm. (**M**) Histopathology scores. *n* = 6 mice per group; experiment repeated 3 times. (**N**) Within-patient comparison of Truelove-Witts scores before and after vancomycin therapy. *n* = 4 patients per group. (**O**) Heatmap represents fecal bile acid (BA) profiling and concentration of 6 BAs of patients receiving vancomycin therapy. *n* = 4 patients per group. Data shown as mean ± SD (**A–O**). Two-sided unpaired *t* test or 2-sided Mann-Whitney *U* test used in **A**, **C**, **E**, **G**, and **H**, depending on data normality. One-way ANOVA with Tukey’s multiple-comparison test or Kruskal-Wallis test with Dunn’s multiple-comparison test used in **I**, **K**, and **M**, based on data normality. Two-sided paired *t* test according to normal distribution in **N** and **O**. Statistical methods and exact *P* values are in Supporting Data Values file. *P < 0.05, **P < 0.01, ***P < 0.001, ****P < 0.0001.

To investigate the mechanisms underlying the clinically observed relief of UC by oral vancomycin administration (2), we used a DSS-induced colitis model, where mice were treated with 2.5% DSS for 5 days, followed by 5 days of recovery (Figure 1A). Compared with vehicle-treated mice, vancomycin promoted colitis recovery, as evidenced by improved weight gain, longer colon length, and reduced histopathological scores (Figure 1, A–E). Targeted metabolomics analysis of fecal metabolites revealed that vancomycin significantly altered the gut metabolome, with 80 differentially regulated metabolites, 65 of which were reduced (Figure 1F; Supplemental Figure 1, A–C; and Supplemental Table 1; supplemental material available online with this article; https://doi.org/10.1172/jci.insight.197470DS1). In line with vancomycin’s inhibition of the genus of *Clostridium*, the abundance of metabolites synthesized by *Clostridium* (18), such as ω-muricholic acid (ω-MCA), hyodeoxycholic acid (HDCA), lithocholic acid (LCA), and DCA, was decreased (Figure 1F). Notably, DCA was one of the most significantly decreased metabolites, with its reduction confirmed in both feces and colon tissue by mass spectrometry (Figure 1, G and H). These findings suggest that DCA may hinder recovery in DSS-induced colitis.

To assess the role of DCA in vancomycin-relieved colitis, we administered a DCA-supplemented diet to vancomycin-treated mice during the recovery phase (Figure 1I). DCA supplementation abolished the promotion of colitis remission by vancomycin, as shown by severe weight loss, shorter colon length, and higher histology scores (Figure 1, I–M). These results indicate that DCA impedes tissue repair, and its elimination by vancomycin ameliorates colitis during the recovery phase.

Clinical evidence demonstrated that vancomycin therapy alleviated colitis in patients with UC (Figure 1N). Mass spectrometry analysis confirmed a significant reduction in fecal DCA levels after vancomycin therapy (Figure 1O). Collectively, the correlation between DCA abundance and vancomycin efficacy in both murine models and humans underscores that the therapeutic effect of vancomycin stems from depletion of DCA.

### DCA impairs colitis remission by inhibiting colonic ILC2 function.

Our previous studies identified that colitis-associated TNF upregulation impairs the bile acid tolerance of intestinal epithelial cells to exacerbate colitis (19). Thus, alleviation of colitis by vancomycin might arise from its impact on bile acid metabolism (18, 20). To this end, we measured the levels of colonic bile acids after vancomycin treatment and observed unaltered colonic bile acids during the recovery phase of DSS-induced colitis (Supplemental Figure 2A).

**Figure 2:**
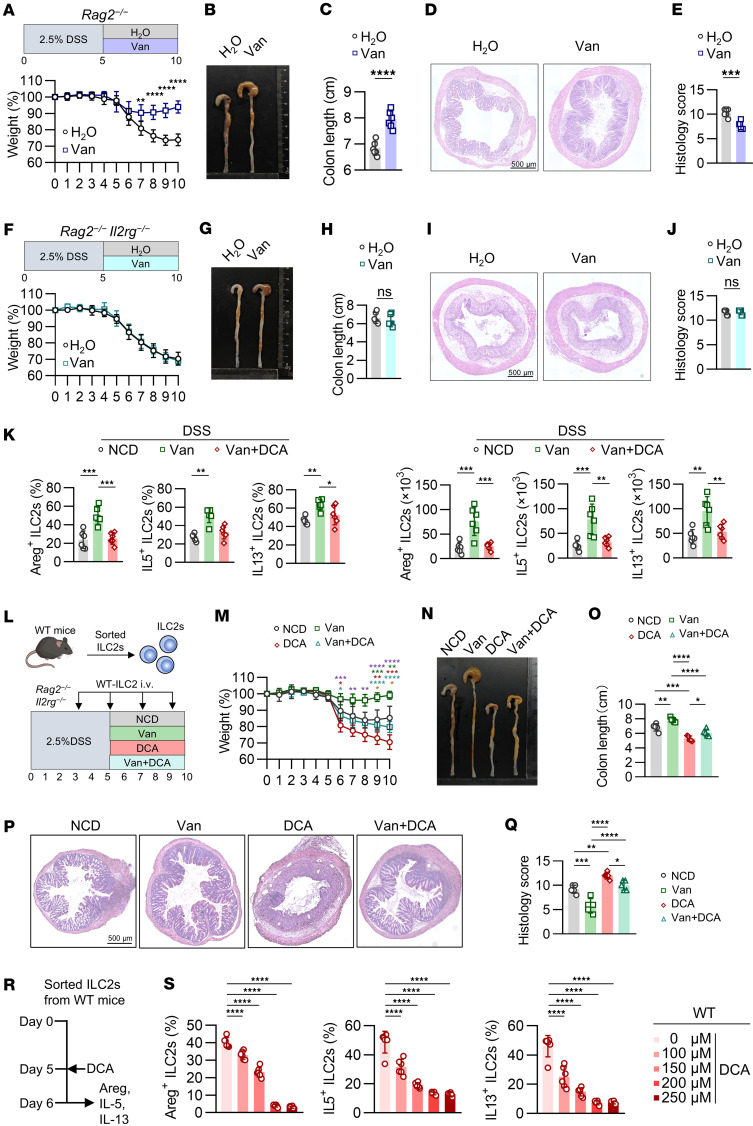
DCA impairs colitis remission by inhibiting colonic ILC2 function. (**A**–**E**) *Rag2*^−/−^ mice received by gavage 5-day H_2_O or vancomycin after 5 days of 2.5% DSS. (**A**) Experimental outline and weight course. (**B**) Representative images and (**C**) colon lengths. (**D**) H&E-stained sections. Scale bar: 500 μm. (**E**) Histopathology scores. *n* = 6 mice per group; experiment repeated 3 times. (**F**–**J**) *Rag2*^−/−^*Il2rg*^−/−^ mice received by gavage 5-day H_2_O or vancomycin after 5 days of 2.5% DSS. (**F**) Experimental outline and weight course. (**G**) Representative images and (**H**) colon lengths. (**I**) H&E-stained sections. Scale bar: 500 μm. (**J**) Histopathology scores. *n* = 6 mice per group; experiment repeated 3 times. (**K**) WT mice received by gavage 5-day H_2_O or vancomycin after 5 days of 2.5% DSS. One group of vancomycin-treated mice were simultaneously fed 2% DCA diet on days 5–10. (left) Percentages and (right) numbers of Areg^+^ ILC2s, IL-5^+^ ILC2s, and IL-13^+^ ILC2s in large intestine. *n* = 6 mice per group; experiment repeated 3 times. (**L**–**Q**) WT LI ILC2s were transferred into *Rag2*^–/–^*Il2rg*^–/–^ mice during colitis recovery. Mice received 2.5% DSS for 5 days, followed by 5-day period of either vancomycin or DCA supplementation. (**L**) Experimental outline. (**M**) Weight course. Green asterisk: NCD vs. Van; red asterisk: NCD vs. DCA; blue asterisk: Van vs. Van+DCA; orange asterisk: DCA vs. Van+DCA; purple asterisk: Van vs. DCA. (**N**) Representative images and (**O**) colon lengths. (**P**) H&E-stained sections. Scale bar: 500 μm. (**Q**) Histopathology scores. *n* = 5 mice per group; experiment repeated twice. (**R** and **S**) Cultured ILC2s were treated with DCA for 24 hours. (**R**) Experimental strategy. (**S**) Percentages of Areg^+^, IL-5^+^, and IL-13^+^ ILC2s. *n* = 6 wells per group; experiment repeated 3 times. Data shown as mean ± SD; 2-sided unpaired *t* test or 2-sided Mann-Whitney *U* test used in **A**, **C**, **E**, **F**, **H**, and **J**, depending on data normality. One-way ANOVA with Tukey’s multiple-comparison test or Kruskal-Wallis test with Dunn’s multiple-comparison test used in **K**, **M**, **O**, **Q**, and **S**, based on data normality. *P < 0.05, **P < 0.01, ***P < 0.001, ****P < 0.0001.

To dissect the cell compartments involved in vancomycin’s relief of colitis, we studied *Rag2*^−/−^ mice lacking T and B cells and *Rag2*^−/−^*Il2rg*^−/−^ mice lacking T and B cells as well as ILCs (21, 22). Compared with vehicle-treated *Rag2*^−/−^ mice, vancomycin promoted colitis recovery, as evidenced by improved weight gain, longer colon length, and reduced histopathological scores (Figure 2, A–E). However, vancomycin lost the ability to promote colitis remission in *Rag2*^−/−^*Il2rg*^−/−^ mice (Figure 2, F–J), suggesting the involvement of ILCs in this process. Given that ILC2s promote epithelial repair and barrier integrity through Areg production (13), we explored whether vancomycin enhances colitis recovery via ILC2s. Flow cytometry revealed that vancomycin treatment increased Areg, IL-5, and IL-13 production in colonic ILC2s of mice with DSS-induced colitis (Supplemental Figure 2, B and C), and DCA supplementation impaired this upregulation (Figure 2K and Supplemental Figure 2D). Consistently, cytometric bead array analysis of colonic tissue lysates showed a reduction in these cytokines after DCA supplementation (Supplemental Figure 2E). To further demonstrate whether vancomycin exerts its therapeutic effect through ILC2s during the recovery phase of colitis, we adoptively transferred colonic ILC2s isolated from WT mice into *Rag2*^–/–^*Il2rg*^–/–^ mice and provided vancomycin-treated mice with a DCA-supplemented diet during the recovery phase (Figure 2L). Compared with vehicle-treated controls, vancomycin-treated mice that received WT-ILC2s showed accelerated weight regain, increased intestinal length, and reduced pathological scores (Figure 2, M–Q). In contrast, DCA supplementation abolished the beneficial effect of vancomycin on colitis remission (Figure 2, M–Q). Collectively, these findings highlight the essential role of ILC2s in mediating the ameliorating effects of vancomycin during colitis recovery.

To further explore vancomycin’s role in ILC2 function, we treated sorted colonic ILC2s with vancomycin in vitro and found no direct effect on their function (Supplemental Figure 2, F and G), suggesting that vancomycin promotes ILC2s indirectly. In contrast, DCA reduced the production of effector molecules in ILC2s in vitro (Figure 2, R and S, Supplemental Figure 3A) and in vivo (Supplemental Figure 3, B and C). Accordingly, DCA inhibited ILC2 function under both physiological and colitis recovery conditions. These findings indicate that vancomycin promotes colitis recovery by enhancing ILC2 function, which is compromised by DCA.

### DCA suppresses colonic ILC2 function via a receptor-independent manner.

**Figure 3:**
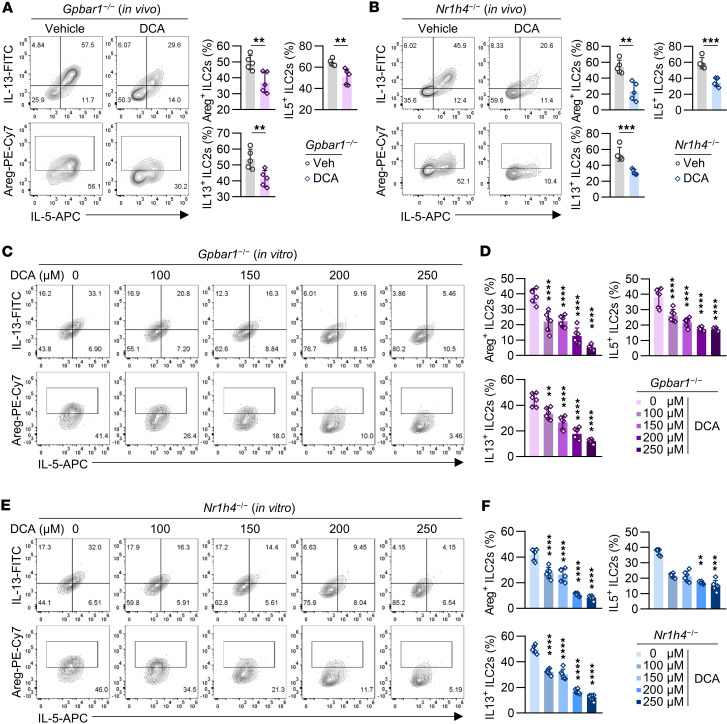
DCA suppresses colonic ILC2 function via a receptor-independent manner. (**A**) *Gpbar1*^–/–^ mice were i.p. injected with DCA or DMSO for 14 days. (**A**, left) Representative flow cytometry plots. (**A**, right) Percentages of Areg^+^, IL-5^+^, and IL-13^+^ ILC2s (CD45.2^+^Lin^–^GATA3^+^) in large intestine. *n* = 5 mice per group. The experiment was repeated twice. (**B**) *Nr1h4*^−/−^ mice were i.p. injected with DCA or DMSO for 14 days. (**B**, left) Representative flow cytometry plots. (**B**, right) Percentages of Areg^+^, IL-5^+^, and IL-13^+^ ILC2s (CD45.2^+^Lin^–^GATA3^+^) in large intestine. *n* = 5 mice per group. The experiment was repeated twice. (**C** and **D**) Large intestinal ILC2s were sorted from *Gpbar1*^–/–^ mice and cultured in the presence of IL-2, IL-7, IL-25, and IL-33 for 5 days. ILC2s were treated with DCA at the indicated concentrations for 24 hours. (**C**) Representative flow cytometry plots. (**D**) Percentages of Areg^+^, IL-5^+^, and IL-13^+^ ILC2s (CD45.2^+^Lin^–^GATA3^+^). *n* = 6 wells per group. The experiment was repeated 3 times. (**E** and **F**) Large intestinal ILC2s were sorted from *Nr1h4*^−/−^ mice and cultured in the presence of IL-2, IL-7, IL-25, and IL-33 for 5 days. ILC2s were treated with DCA at the indicated concentrations for 24 hours. (**E**) Representative flow cytometry plots. (**F**) Percentages of Areg^+^, IL-5^+^, and IL-13^+^ ILC2s (CD45.2^+^Lin^–^GATA3^+^). *n* = 6 wells per group. The experiment was repeated 3 times. Data are shown as mean ± SD; 2-sided unpaired *t* test or 2-sided Mann-Whitney *U* test was used in **A** and **B**, depending on data normality. One-way ANOVA with Tukey’s multiple-comparison test or Kruskal-Wallis test with Dunn’s multiple-comparison test was used in **D** and **F**, based on data normality. Statistical methods and exact *P* values are provided in the Supporting Data Values file. **P < 0.01, ***P < 0.001, ****P < 0.0001.

DCA usually functions as a ligand through activating its receptors, such as TGR5 and FXR (23). To determine whether these receptors mediate DCA’s effects on ILC2s, we treated *Gpbar1^−/−^* (TGR5-KO) and *Nr1h4^−/−^* (FXR-KO) mice with DCA. Despite the absence of these receptors, DCA still impaired colonic ILC2 function in both strains (Figure 3, A and B). Additionally, when sorted ILC2s from these mice were treated with DCA in vitro, Areg, IL-5, and IL-13 production were reduced (Figure 3, C–F), confirming that DCA’s inhibitory effects on ILC2s are independent of TGR5 and FXR. To further assess whether TGR5 and FXR signaling exert cell-intrinsic effects in ILC2s, we generated mixed bone marrow chimeras in *Rag2*^–/–^*Il2rg*^–/–^ mice using WT (CD45.1) together with either *Gpbar1*^−/−^ (CD45.2) or *Nr1h4*^−/−^ (CD45.2) bone marrow cells. After 4 weeks of reconstitution, mice received a 2-week DCA treatment (Supplemental Figure 4A). Subsequent analysis showed that colonic ILC2s and ILC2s derived from WT donors producing Areg, IL-5, and IL-13 were comparable to ILC2s originating from either *Gpbar1*^−/−^ or *Nr1h4*^−/−^ bone marrow (Supplemental Figure 4, B–E).

We further evaluated whether DCA-induced inhibition of ILC2 function was due to cell death, given bile acids’ detergent properties. DCA reduced the production of effector molecules by ILC2, but it did not affect cell viability (Supplemental Figure 4F). In contrast, SDS, which has similar cytotoxicity to DCA (24), did not reduce cytokine production in ILC2s to the same extent (Supplemental Figure 4, G and H). These results suggest that DCA inhibits ILC2 function without inducing cell death via its detergent-like activity.

### DCA disrupts protein folding in ILC2s by binding to TMX2 in the ER.

**Figure 4:**
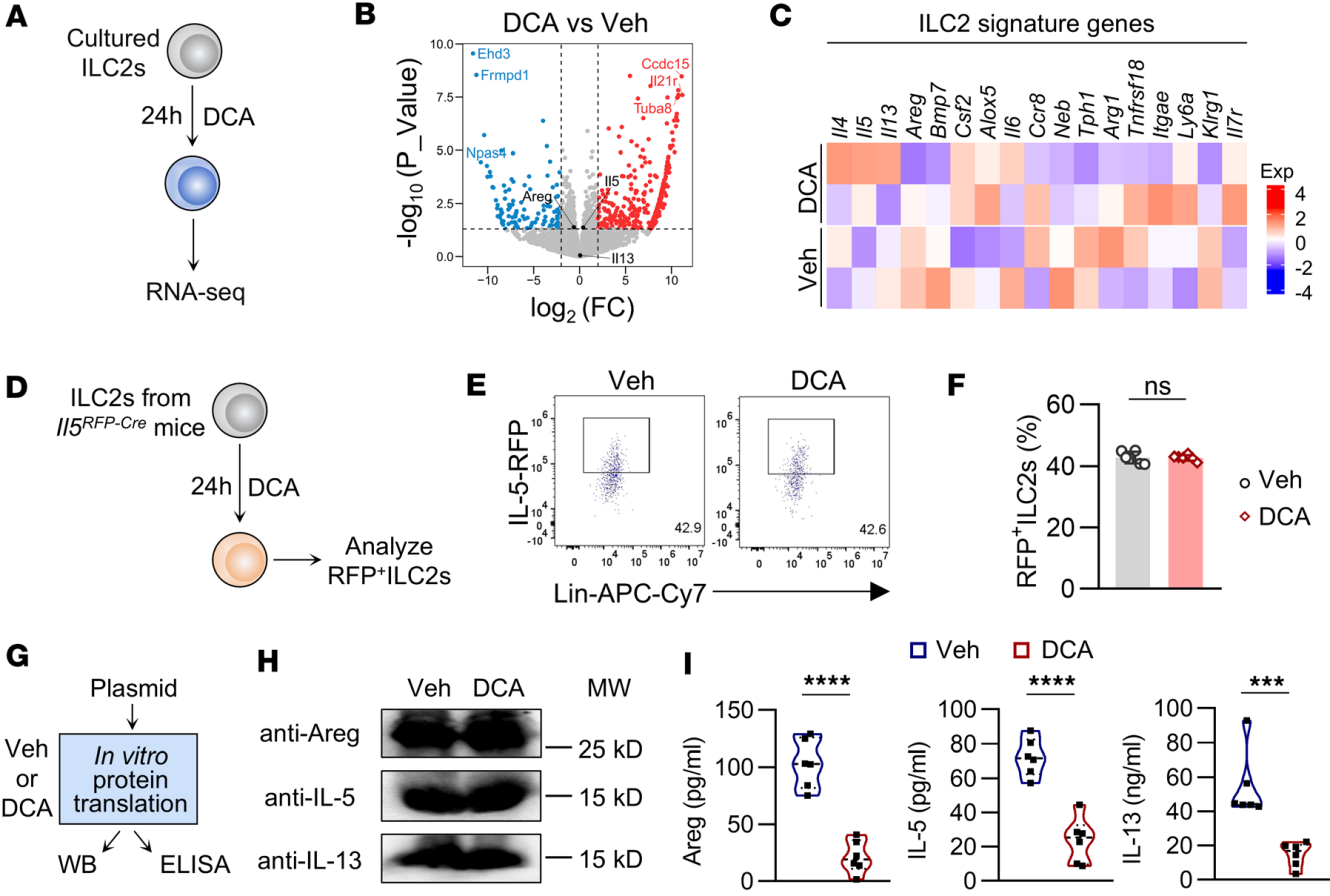
Inhibition of DCA on ILC2 function does not occur at transcription or translation. (**A**–**C**) Large intestinal ILC2s were sorted from WT mice and cultured in the presence of IL-2, IL-7, IL-25, and IL-33 for 5 days. ILC2s were treated with DCA (100 μM) at the indicated concentrations for 24 hours and then subjected to RNA-Seq. (**A**) Experimental strategy, (**B**) volcano plot, and (**C**) heatmap of ILC2-characteristic genes. (**D**–**F**) Sorted large intestinal ILC2s from *Il5^RFP-Cre^* mice were treated with DCA (100 μM) for 24 hours. (**D**) Experimental strategy. (**E**) Representative flow cytometry plots. (**F**) Percentages of RFP^+^ ILC2s (CD45.2^+^Lin^–^GATA3^+^) in large intestine. *n* = 6 wells per group. The experiment was repeated 3 times. (**G**–**I**) Rabbit reticulocyte lysate system. (**G**) Experimental strategy. Protein levels of Areg, IL-5, and IL-13 were analyzed by (**H**) Western blotting and (**I**) cytometric bead array in samples treated or untreated with DCA. *n* = 6 wells per group. The experiment was repeated twice. Data are shown as mean ± SD. Statistical analysis was performed using unpaired 2-tailed *t* test. ***P < 0.001, ****P < 0.0001.

To understand how DCA inhibits ILC2 function, we performed RNA-Seq on sorted ILC2s treated with DCA in vitro. DCA did not alter the transcription of characteristic ILC2 genes, including *Il5*, *Il13*, and *Areg* (Figure 4, A–C). In addition, sorted ILC2s from *Il5*^RFP-Cre^ mice treated with DCA showed that the RFP signals in ILC2s, which reflected the transcription of *Il5*, were not altered by DCA (Figure 4, D–F). These findings suggest the inhibition of DCA on ILC2s occurs posttranscriptionally. Further analysis using an in vitro protein synthesis system revealed that DCA had no impact on the translation of *Areg*, *Il5,* and *Il13*, the effector molecules of ILC2s (Figure 4, G and H). However, cytometric bead array analysis revealed that DCA treatment significantly reduced the protein levels of these cytokines in ILC2s (Figure 4I), indicating that DCA inhibited the protein processing of these secretory proteins posttranslationally.

**Figure 5:**
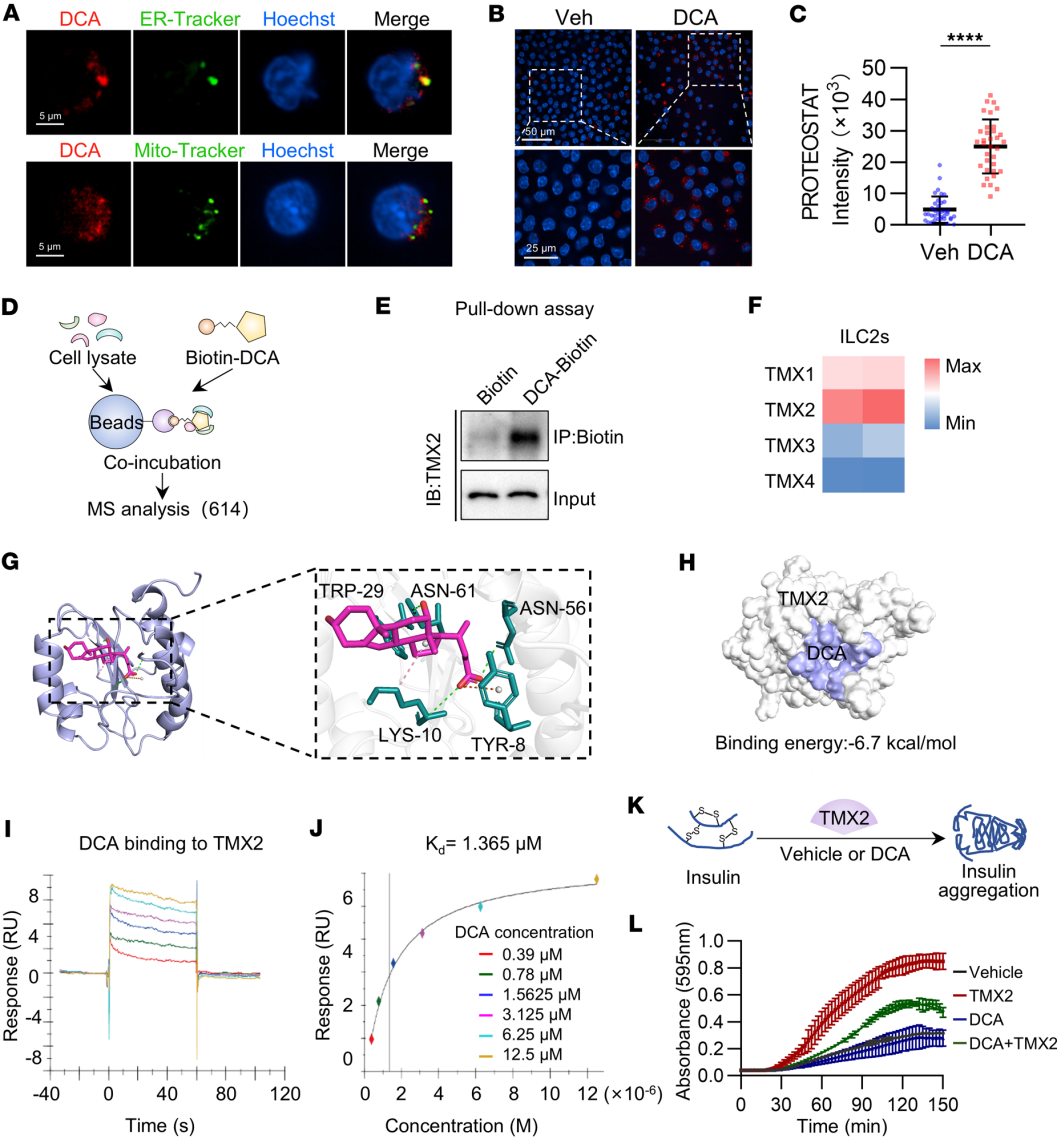
DCA disrupts protein folding in ILC2s by binding to TMX2 in the ER. (**A**) ILC2s were treated with DCA-iF647 (1 μM, red) for 15 minutes, followed by staining with ER-Tracker (green) or Mito-Tracker (green), and Hoechst 33342 (blue). Representative confocal laser scanning microscopy (LSCM) images. (**B** and **C**) Protein misfolding was quantified using the molecular rotor Proteostat with affinity for aggregated proteins. Increased protein aggregation causes the dye to stop spinning and emit fluorescence. We analyzed misfolding in ILC2s treated with DCA (100 μM) for 24 hours. (**B**) Representative confocal LSCM images. (**C**) Relative mean intensity per cell was plotted and calculated after blinding. *n* = 36 for DMSO-treated ILC2s, *n* = 34 for ILC2s treated with 100 μM DCA. The experiment was repeated 3 times. (**D**) Schematic of DCA-protein pulldown assays in cell lysates in combination with mass spectrometry analysis. (**E**) Representative immunoblot of TMX2 in proteins pulled down from ILC2 lysates by biotin-DCA (100 μM). (**F**) Heatmap of TMX family genes by performing RNA-Seq analysis with purified ILC2s. (**G** and **H**) Molecular docking showing the binding of DCA to TMX2. (**I** and **J**) Surface plasmon resonance assay for the affinity between different concentrations of DCA and purified TMX2 protein. RU, resonance unit. (**K** and **L**) Reductase activity of TMX2. (**K**) Experimental strategy. (**L**) Purified TMX2 protein with or without DCA (200 μM) was incubated with insulin, and the ability to reduce insulin disulfide bonds was measured. The absorbance at 595 nm was monitored every 2.5 minutes. *n* = 3 wells per group. Data shown as mean ± SD. Statistical analysis was performed using unpaired 2-tailed *t* test. ****P < 0.0001.

The secretory proteins acquired specific 3D structures in the ER to become functional and were then secreted out of the cells. In accordance, our confocal imaging analysis revealed that DCA localized to the ER in ILC2s, revealed by colocalization of DCA-iF647 and ER-Tracker (Figure 5A). Furthermore, the use of fluorescent dye–based reagent Proteostat, which exhibits red fluorescent signals binding to misfolded proteins, showed a remarkable increase of protein misfolding in ILC2s treated with DCA (Figure 5, B and C). To uncover how DCA affects protein processing in the ER, a pulldown assay using biotin-labeled DCA identified 614 DCA-interacting proteins, of which TMX2, a protein with disulfide reductase activity in the ER, was of particular interest (Figure 5, D and E, and Supplemental Table 4). The TMX protein family consists of TMX1, TMX2, TMX3, and TMX4, with TMX2 exhibiting the highest level of expression in ILC2s (Figure 5F). The interaction between DCA and TMX2 was corroborated by immunoblotting in a DCA pulldown assay with ILC2 lysates (Figure 5E), and molecular docking analysis showed a high binding affinity, with a binding energy of –6.7 kcal/mol (Figure 5, G and H). Surface plasmon resonance assay further supported that DCA directly binds to TMX2 with a dissociation constant, Kd, at 1.365 μM (Figure 5, I and J).

To further confirm that DCA impaired protein processing, commonly as protein misfolding, by interacting with TMX2, an insulin disulfide reducing assay was performed to analyze the disulfide reductase activity of TMX2 in the presence of DCA (Figure 5K). The results showed that TMX2 acted as a disulfide reductase, which was inhibited by DCA (Figure 5L).

### The PERK/eIF2α branch of UPR mediates the inhibitory effects of DCA on ILC2 function and colitis recovery.

**Figure 6:**
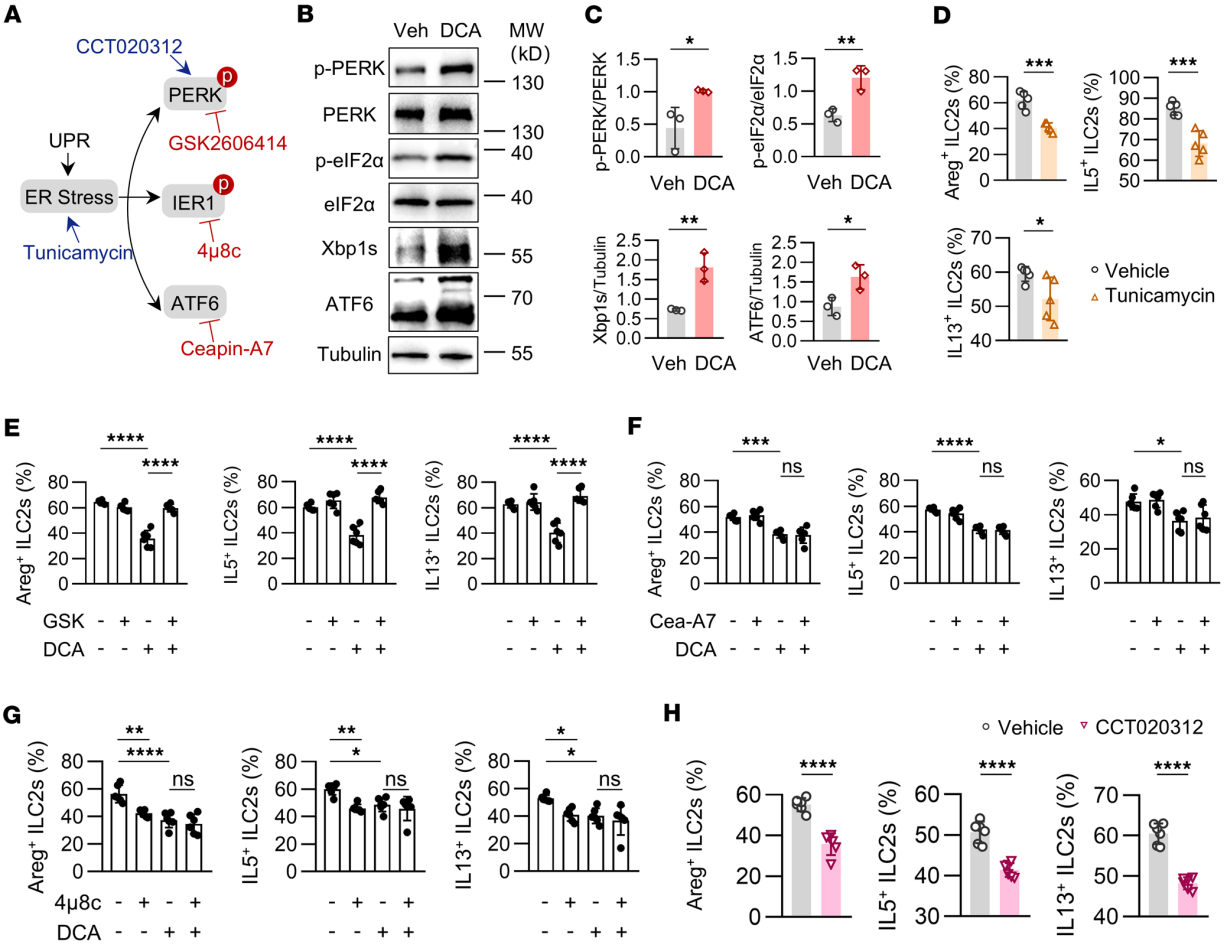
DCA enhances UPR in ILC2s. Large intestinal ILC2s were sorted from WT mice and cultured in the presence of IL-2, IL-7, IL-25, and IL-33 for 5 days. (**A**) Schematic representation of the ER stress signaling pathways. (**B** and **C**) ILC2s were treated with 100 μM DCA for 24 hours. (**B**) Immunoblotting of p-PERK, PERK, p-eIF2α, eIF2α, Xbp1s, ATF6. (**C**) Expression levels of p-PERK/PERK, p-eIF2α/eIF2α, Xbp1s, and ATF6 in ILC2s. p-PERK, phospho-PERK; eIF2α, eukaryotic initiation factor-2α; p-eIF2α, phospho-eIF2α; Xbp1s, spliced X-box binding protein-1; ATF6, activating transcription factor 6. (**D**) ILC2s were treated with ER stress activator (tunicamycin, 0.3 μM) for 24 hours. Percentages of Areg^+^, IL-5^+^, and IL-13^+^ ILC2s (CD45.2^+^Lin^–^GATA3^+^). *n* = 5 wells per group. The experiment was repeated twice. (**E**–**G**) Percentages of Areg^+^, IL-5^+^, and IL-13^+^ ILC2s (CD45.2^+^Lin^–^GATA3^+^) in groups treated with ER stress inhibitors targeting (**E**) PERK phosphorylation (GSK, 10 μM), (**F**) ATF6 (Cea-A7, 10 μM), and (**G**) IRE1α (4μ8c, 20 μM). ILC2s were treated with DMSO or DCA (100 μM) for 24 hours. *n* = 6 wells per group. The experiment was repeated 3 times. (**H**) Percentages of Areg^+^, IL-5^+^, and IL-13^+^ ILC2s (CD45.2^+^Lin^–^GATA3^+^) in groups treated with PERK phosphorylation activator (CCT020312, 10 μM). *n* = 6 wells per group. The experiment was repeated 3 times. Data shown as mean ± SD; 2-sided unpaired *t* test or 2-sided Mann-Whitney *U* test used in **C**, **D**, and **H**, depending on data normality. One-way ANOVA with Tukey’s multiple-comparison test or Kruskal-Wallis test with Dunn’s multiple-comparison test used in **E**–**G**, based on data normality. Statistical methods and exact *P* values are in the Supporting Data Values file. *P < 0.05, **P < 0.01, ***P < 0.001, ****P < 0.0001.

The accumulation of misfolded proteins in the ER triggers the UPR (25), which activates 3 pathways, IRE1α/Xbp1, PERK/eIF2α, and ATF6 (Figure 6A). We observed that DCA treatment increased the levels of spliced Xbp1, phosphorylated PERK and eIF2α, and ATF6 in ILC2s (Figure 6, B and C). Similarly, treatment with tunicamycin, an ER stress inducer, impaired ILC2 function, further highlighting the detrimental effect of ER stress on gut ILC2s (Figure 6D and Supplemental Figure 5A). To identify the specific UPR pathway responsible for DCA-induced ILC2 dysfunction, we treated sorted ILC2s with DCA in combination with inhibitors targeting each UPR branch. Only inhibition of PERK phosphorylation by GSK2606414 restored ILC2 function (Figure 6, E–G, and Supplemental Figure 5, B–D). Consistently, CCT020312, a PERK phosphorylation agonist, suppressed effector molecule production by ILC2s (Figure 6H and Supplemental Figure 5E).

**Figure 7:**
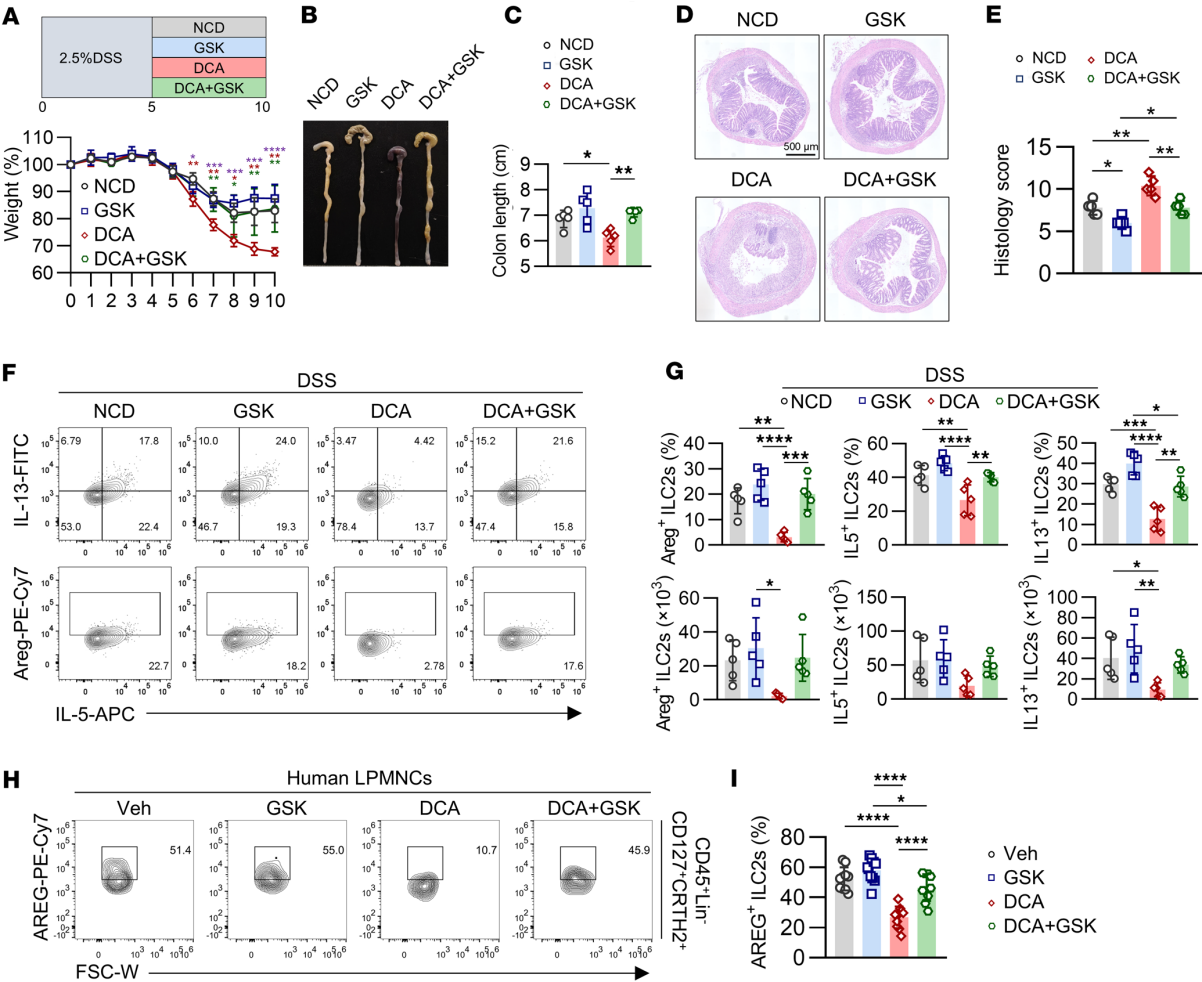
The PERK branch of UPR mediates DCA-inhibited ILC2 function and colitis recovery. (**A**–**G**) WT mice received NCD or DCA by gavage for 5 days, starting 5 days after 2.5% DSS administration. One group of NCD- or DCA-fed mice was simultaneously i.p. injected with GSK2606414 or vehicle for 5 days. (**A**, top) Experimental outline is shown. (**A**, bottom) Weight course. The red asterisk represents comparison between NCD and DCA groups; green asterisk represents comparison between DCA and DCA+GSK groups; purple asterisk represents comparison between Van and DCA groups. (**B**) Representative image of colon. (**C**) Statistical data of colon length. (**D**) H&E-stained histological sections. Scale bar: 500 μm. (**E**) Histopathology scores. (**F**) Representative flow cytometry plots. (**G**, top) Percentages of Areg^+^, IL-5^+^, and IL-13^+^ ILC2s (CD45.2^+^Lin^–^GATA3^+^) and (**G**, bottom) absolute numbers of Areg^+^ ILC2s, IL-5^+^ ILC2s, and IL-13^+^ ILC2s in large intestine. *n* = 5 mice per group. The experiment was repeated twice. (**H** and **I**) Human colonic lamina propria mononuclear cells were stimulated with DCA at 100 μM, supplemented with or without GSK2606414 for 16 hours in complete RPMI 1640 media. (**H**) AREG expression was analyzed after gating on CD45^+^Lin^−^CD127^+^CRTH2^+^ ILC2s. (**I**) Percentage of AREG^+^ ILC2s. Connected lines are samples from the same clinical samples (*n* = 10). Data are shown as mean ± SD. One-way ANOVA with Tukey’s multiple-comparison test or Kruskal-Wallis test with Dunn’s multiple-comparison test used in **A**, **C**, **E**, **G**, and **I**, based on data normality. Statistical methods and exact *P* values are provided in the Supporting Data Values file.*P < 0.05, **P < 0.01, ***P < 0.001, ****P < 0.0001.

To explore the therapeutic potential of targeting the PERK/eIF2α axis of UPR, DCA-treated mice were administered GSK2606414 during the recovery phase of DSS-induced colitis. Inhibition of PERK phosphorylation facilitated colitis remission, as evidenced by improved weight gain, colon length, and histological scores (Figure 7, A–E). Flow cytometry revealed enhanced production of Areg, IL-5, and IL-13 in colonic ILC2s, suggesting that PERK activation impedes tissue repair during colitis recovery (Figure 7, F and G). In accordance, DCA treatment of human lamina propria mononuclear cells significantly reduced AREG expression in human colonic ILC2s, and this inhibition was restored by GSK2606414 (Figure 7, H and I). Moreover, ISRIB, a PERK/eIF2α inhibitor that restores eIF2α phosphorylation, also rescued DCA-inhibited ILC2 function and promoted tissue repair (Supplemental Figure 6, A–J). These findings indicate that the PERK/eIF2α arm of the UPR mediates the inhibitory effect of DCA on ILC2s and impedes colitis recovery.

## Discussion

Our data revealed a mechanism by which vancomycin promotes colitis remission via regulation of the microbiota-derived metabolite DCA. Although antibiotic therapy remains excluded from routine UC treatment protocols due to inadequate clinical efficacy (26–29), recent studies demonstrate that oral vancomycin exerts therapeutic benefits by remodeling microbial communities and associated metabolic pathways (2, 4, 30, 31). In contrast, i.p. administration rather than the oral route exhibits paradoxical effects in that vancomycin compromises goblet cell mucus production and intensifies intestinal inflammation through microbiota-independent mechanisms (32). This contradiction might underscore the critical role of gut microbiota in vancomycin’s therapeutic effects on UC, since the i.p. delivery of vancomycin has a restricted ability to modulate gut microbiota. These results highlight the importance of precisely targeting the gut microbiota in therapies for UC.

DCA is a secondary bile acid synthesized by gut microbiota (33–36). Our study demonstrates that DCA exacerbates DSS-induced experimental colitis by suppressing ILC2 function. However, some studies suggest that secondary bile acids may promote intestinal stem cell proliferation and tissue repair through TGR5 signaling in the acute phase of DSS-induced colitis (37, 38). This discrepancy may arise from the negligible expression of TGR5 on ILC2s, and our experimental evidence indicates that DCA retains inhibitory effects on ILC2s even in *Gpbar1^−/−^* mice (Figure 3, A, C, and D). Accordingly, bile acids, particularly those with high hydrophobicity such as DCA (39), passively diffuse into cells owing to their detergent-like properties (35, 40–42), ultimately accumulating in the ER (19). We further confirm that DCA impairs the pro-repair function of ILC2s by binding to the ER transmembrane protein TMX2, thereby disrupting the UPR. Furthermore, compared with the oral administration of DCA used in our study, prior research employing rectal delivery might result in lower DCA concentrations and shorter exposure durations, likely leading to the opposite observations.

Currently, the clinical use of vancomycin in UC is primarily considered for patients with concomitant primary sclerosing cholangitis, a subgroup characterized by an abnormally elevated abundance of Gram-positive cocci (43, 44), which are the target of vancomycin (45, 46). Notably, the patients enrolled in our study presented with an abnormal cocci-to-bacilli ratio (9:1), which is typically an indication for vancomycin treatment. Thus, it should be emphasized that vancomycin may be suitable for a specific UC subpopulation, despite its broader activity against Gram-positive bacilli, including *Clostridium* species, which are primary producers of secondary bile acids such as DCA.

Our prior work demonstrates that bile acids exacerbate colitis as TNF compromises the detoxification of bile acids in intestinal epithelial cells (19). Although the findings of this study revealed that vancomycin promoted colitis remission through elimination of DCA, we noticed that vancomycin functions to remodel gut microbial communities (47–50), specifically suppressing the bioconversion of cholic acid to DCA (8, 36, 51–54) without altering total bile acid pools. Notably, vancomycin treatment paradoxically lost therapeutic efficacy in *Rag2*^–/–^*Il2rg*^−/−^ mice (Figure 2, F–J), unequivocally demonstrating the indispensable role of ILCs in vancomycin-promoted colitis recovery. Of critical pathophysiological relevance, we identified DCA, rather than cholic acid, as a potent selective suppressor of ILC2 function through disrupting the proteostasis in these cells (data not shown). These results establish a transformative strategy targeting the microbiota/DCA/ILC2 axis, whereby vancomycin-mediated remodeling of gut microbial bile acid metabolism reverses DCA-induced ILC2 dysfunction. Consistently, in patients with UC treated with oral vancomycin, we observed a reduction in DCA levels accompanied by alleviation of colitis. Nonetheless, the limited sample size in the patient cohort represents a constraint, and future studies with larger patient populations will be essential to further validate these observations.

## Methods

### Sex as a biological variable.

Our study examined male and female animals, and similar findings are reported for both sexes.

### Study design.

This study aimed to explore whether and how ILC2s are altered within the colonic tissues during colitis. To address this question, we used metabolomics, RNA-Seq, and flow cytometry analyses to uncover the important role of microbiota-derived DCA in ILC2. All mice were housed in specific pathogen–free conditions with ad libitum food and water and a 12-hour light/12-hour dark cycle at the Model Animal Research Center of Shandong University. For the experiments, 6- to 14-week-old male or female mice were used for the experiments. Experimental parameters, animal numbers, experimental replicates, and statistical approaches are listed in corresponding figure legends and later in the Methods.

### Patients.

A total of 4 patients with UC participated in this study. All patients with UC were recruited from the Department of Gastroenterology, Qilu Hospital of Shandong University. The diagnosis of UC was based on well-established clinical, endoscopic, and histopathological criteria by experienced physicians. Exclusion criteria were as follows: combined with Crohn’s disease; immunosuppressive and hormone therapy within the past 2 months; combined with severe infection, malignant tumor, or liver or kidney insufficiency; pregnant or breastfeeding; and other causes of intestinal inflammation, such as infectious enteritis and amebic colitis. We used the Truelove-Witts score, which is based on the Japanese Society of Gastroenterology’s scoring criteria, to assess clinical disease activity. Two experienced gastroenterologists independently reviewed the medical records of patients, and the patients were enrolled in the study only when both experts agreed on the scores. In cases of disagreement between gastroenterologists on scores, a third expert was asked to give a final opinion. The clinical characteristics of the patients and basic information including age, sex, weight, and BMI are provided in Supplemental Table 2, and basic information including age, sex, weight, and BMI is in Supplemental Table 2. Stool samples were collected from 4 vancomycin-treated patients with UC who were not receiving any other targeted therapy. All human stool samples were stored at –80°C for further metabolomics analysis. Human colonic mucosal biopsies were obtained at Qilu Hospital of Shandong University. The human colonic mucosal biopsy samples were obtained from 40 healthy donors. Exclusion criteria of healthy donors were as follows: had polyps larger than 1.0 cm; had intestinal inflammation; combined with malignant tumor. The demographic information of healthy donors is listed in Supplemental Table 3. The experimental protocols were performed according to the guidelines approved by the Ethics Committee of Shandong University (ECSBMSSDU2020-1-035).

### Mice.

Male and female C57BL/6J mice at the age of 6–8 weeks old were used for all animal experiments. Specific pathogen–free C57BL/6J mice, *Nr1h4*-KO mice (background: C57BL/6J, strain T012640), and *Gpbar1*-KO mice (background: C57BL/6J, strain T012754) were purchased from GemPharmatech. *Rag2*^−/−^ mice and *Rag2*^−/−^*Il2rg*^−/−^ mice (catalog 4111) were purchased from Taconic Biosciences. *Il5*^RFP-Cre^ mice (catalog R5/+) and CD45.1/CD45.1 mice (catalog 002014) were purchased from The Jackson Laboratory. Transgenic mice and WT mice were bred and maintained within sterile isolators at the laboratory animal center of Shandong University on a 12-hour light/12-hour dark cycle with ambient temperature of 20°C–26°C, humidity of 40%–70%, and ad libitum access to food and water. Both male and female mice with no previous history of experiments were used, except for C57BL/6J mice, which used only males. All the animal experiments were approved by the IACUC of Shandong University (ECSBMSSDU2020-2-057).

### Diets.

Normal control diet (AIN93G) mice were fed standard mouse chow based on the formula of a purified diet for experimental rodents published by the American Institute of Nutrition in 1993. All bile acid–containing diets used were synthesized at a concentration of 0.2% (weight/weight) based on NCD. All experimental diets used in this study were purchased (NCD) from or synthesized (DCA diet) by Jiangsu Xietong Pharmaceutical Bio-engineering Co., Ltd.

### DSS-induced colitis model.

DSS was purchased from MP Biomedicals. Mice were treated with 2.5% (w/v) DSS in drinking water for 5 days as indicated. Body weight was recorded by the same operator at the same time each day to determine the percentage change in body weight. When mice exhibited clear symptomatic monitoring criteria (including a 20% reduction in body weight compared with pre-experimental weight, inability to eat or drink, abnormal posture, being moribund or immobile, or no response to gentle stimulation), they were euthanized. Colon length was measured. The middle part of the colon tissue was fixed with 4% formalin, embedded in paraffin, sectioned, and stained with H&E.

### Dietary treatments.

For experiments investigating the effect of DCA on the remission of colitis, mice received either DCA for 5 days, before which the drinking water was replaced with 2.5% DSS (w/v) for 5 days. Mice fed with antibiotics were treated in the same way.

### Drug administration.

For experiments investigating the effects of antibiotics on the remission of colitis, mice were treated with vancomycin 0.5 g/L daily for 5 days after a 5-day period of DSS administration, and drinking water was provided instead of DSS during vancomycin treatment. For DCA treatment, mice were given 25 mM DCA at 100 μL daily by i.p. injection throughout the experiment, starting from day –14. For GSK2606414 treatment in vivo experiments, DSS was withdrawn on day 5, and mice were subsequently treated once daily with 100 μL vehicle (10% DMSO, 40% PEG 400, and 5% Tween 80 in distilled water) or GSK2606414 at a dose of 5 mg/kg body weight by i.p. injection. Mice were euthanized and large intestine was collected for analysis of ILC2 effector function on day 10. For in vivo experiments examining ISRIB treatment, DSS was withdrawn on day 5, and mice were subsequently treated once daily with 100 μL vehicle (10% DMSO, 40% PEG 400, and 5% Tween 80 in distilled water) or ISRIB at a dose of 2.5 mg/kg body weight by i.p. injection. Mice were euthanized and large intestine was collected for analysis of ILC2 effector function on day 10.

### Histopathology.

The colon tissue collected from the same region (mid-colon) was harvested and placed into 4% paraformaldehyde immediately and kept at room temperature for 24 hours before a standardized dehydration procedure. Then, dehydrated colon tissue was embedded in paraffin and cut into 4 μm sections, which were stained with H&E. Stained sections were imaged by Olympus VS120 Slide Scanner. For pathological assessment, all evaluators were blind to the grouping of histological sections and assigned scores based on tissue inflammation (0–5), crypt damage (0–4), ulceration (0–3), and edema (0–1), which were then recorded by another researcher.

### Immunofluorescence.

For experiments investigating the intracellular localization of DCA and the ER, cells seeded in confocal dishes were incubated with DCA-iF647 for 30 minutes, followed by nuclei and ER or mitochondria staining using Hoechst 33342 (Beyotime) and ER-Tracker Green (Beyotime) or Mito-Tracker Green (Beyotime) for 30 minutes. Cells in confocal dishes were washed 3 times with RPMI 1640 and imaged.

### Isolation of immune cells from intestinal lamina propria lymphocytes.

The isolation of mouse immune cells from intestinal lamina propria lymphocytes was done as previously described (55). Briefly, large intestines were separated and fat tissues were removed. Intestines were cut open and washed in PBS, and were then cut into pieces 1 cm long, washed, and shaken in PBS for 2 minutes. Intestines were incubated in PBS containing 30 mm EDTA and 10 mm HEPES with shaking at 200 rpm at 37°C for 30 minutes. The tissues were then digested in RPMI 1640 containing FBS (5%), 1% penicillin-streptomycin, DNase I (150 μg/mL, Sigma-Aldrich), and collagenase VIII (150 U/mL, Sigma-Aldrich) at 37°C in a 5% CO_2_ incubator for 1.5 hours. The digested tissues were shaken and filtered through 100 μm cell strainers. Mononuclear cells were then harvested from the interphase of an 80% and 40% Percoll (Cytiva) gradient after a spin at 1,335*g* for 15 minutes at room temperature. For the isolation of human lamina propria mononuclear cells, the mucosal tissues were washed twice in PBS, and then incubated in PBS containing 10 mM EDTA and 5% FBS at 37°C with shaking at 220 rpm for 20 minutes. After washing once in RPMI 1640 medium, the mucosal tissues were digested for 30 minutes in RPMI 1640 medium containing 5% FBS, 1% penicillin-streptomycin, DNase I (150 μg/mL), and collagenase IV (5 mg/mL, C5138; Sigma-Aldrich) with shaking at 80 rpm at 37°C. The digested tissues were shaken and dissociated into single cells and filtered through a 100 μm cell strainer. After centrifugation, the mononuclear cell pellets were harvested. Samples from the 4 individuals were pooled and treated as a single biological replicate, with each replicate receiving drug treatment at the indicated concentrations.

### Flow cytometry and cell sorting.

The live and dead cells were discriminated by Aqua Fixable Viability kit (BioLegend) in PBS. CD16/32 antibody (eBioscience) was used to block the nonspecific binding to Fc receptors before surface staining. For cell surface staining, cells were stained with surface-labeled antibodies for 25 minutes at 4°C. For intracellular staining, cells were incubated with fixation/permeabilization. After fixation, the cells were incubated with the indicated antibodies for 2 hours at 4°C. For cytokine production, cells were cultured in complete IMDM medium and stimulated by 50 ng/mL PMA and 500 ng/mL ionomycin for 4 hours, and Brefeldin A (2 μg/mL) was added 2 hours before cells were harvested. Lineage-positive cells were excluded from the analysis of ILCs by staining for the mix (Lin) containing CD3, CD5, CD19, B220, Ly6G, CD11b, CD11c, Ter119, FcεRIα, and CD16/32. Flow cytometry was performed using the Gallios flow cytometer (Beckman Coulter) and analyzed with FlowJo software. Cell sorting was performed on MoFlo Astrios EQS (Beckman Coulter) or BD FACSMelody (BD Biosciences). ILC2s were sorted as the CD45.2^+^Lin^–^CD127^+^KLRG1^+^ population from mouse large intestinal lamina propria lymphocytes. For a detailed list of all antibodies used, refer to Supplemental Table 5.

### Cell culture.

Immune cells from intestinal lamina propria were cultured in IMDM media (Macgene Biotechnology) supplemented with 5% FBS for 4 hours, or in IMDM media supplemented with 10% FBS for 24 hours. Sorted ILC2s were cultured in IMDM media supplemented with 15% FBS, mIL-2 (10 ng/mL, PeproTech), mIL-7 (10 ng/mL, PeproTech), mIL-25 (10 ng/mL, PeproTech), and mIL-33 (10 ng/mL, PeproTech) for indicated times. Human lamina propria mononuclear cells were cultured in RPMI 1640 medium (CM10040; Macgene Biotechnology) supplemented with 10% FBS for the indicated times. All media contained 1% penicillin/streptomycin, 0.1% gentamycin, and the cells were maintained at 37°C in a 5% CO_2_ incubator.

### Adoptive transfer of ILC2s.

For adoptive transfer of ILC2s under colitis, large intestinal ILC2s from WT mice were cultured in complete IMDM medium containing IL-2, IL-7, and IL-25, and IL-33 (10 ng/mL each) for 5 days. A total of 1 × 10^7^ ILC2s were transferred into *Rag2*^–/–^*Il2rg*^–/–^ mice on days 3, 5, 7, and 9, after which the mice were administered 2.5% DSS (w/v) in drinking water for 5 days to induce colitis, followed by a 5-day recovery period. Mice were treated with either a DCA diet or vancomycin in their drinking water beginning on day 5 and were analyzed at day 10.

### Metabolomics assay.

For fecal sample preparation, samples were accurately weighed and put into a 2 mL centrifuge tube with 1,000 μL tissue extract (75% 9:1 methanol/chloroform, 25% H_2_O), and 3 steel balls were added. Samples were ground twice at 50 Hz for 60 seconds, exposed to ultrasound for 30 minutes at room temperature, and incubated on ice for 30 minutes. Tubes were centrifuged for 10 minutes at 13,523*g*, 4°C, and the supernatant was transferred into a new 2 mL centrifuge tube, concentrated, and dried. Next, 200 μL 50% acetonitrile solution prepared with 2-amino-3-(2-chloro-phenyl)-propionic acid (4 ppm) was added to re dissolve the samples. Then, the supernatant was filtered through a 0.22 μm membrane into the detection bottle for liquid chromatography–mass spectrometry (LC-MS) detection.

### LC conditions.

LC analysis was conducted using a Vanquish UHPLC system (Thermo Fisher Scientific) equipped with an ACQUITY UPLC HSST3 column (2.1 × 150 mm, 1.8 μm, Waters). The flow rate was set to 0.25 mL/min, with a column temperature of 40°C and an injection volume of 2 μL.

For the positive ion mode, the mobile phase consisted of 0.1% formic acid in acetonitrile (C) and 0.1% formic acid in water (D). The gradient elution program was as follows: 0–1 minute, 2% C; 1–9 minutes, 2%–50% C; 9–12 minutes, 50%–98% C; 12–13.5 minutes, 98% C; 13.5–14 minutes, 98%–2% C; 14–20 minutes, 2% C.

For the negative ion mode, the mobile phase comprised acetonitrile (A) and 5 mM ammonium formate in water (B). The gradient elution program was 0–1 minute, 2% A; 1–9 minutes, 2%–50% A; 9–12 minutes, 50%–98% A; 12–13.5 minutes, 98% A; 13.5–14 minutes, 98%–2% A; 14–17 minutes, 2% A.

### MS conditions.

MS detection of metabolites was performed on Orbitrap Exploris 120 (Thermo Fisher Scientific) with ESI ion source. Simultaneous MS1 and MS/MS (Full MS-ddMS2 mode, data-dependent MS/MS) acquisition was used. The parameters were as follows: sheath gas pressure, 30 arb; aux gas flow, 10 arb; spray voltage, 3.50 kV and –2.50 kV for ESI (+) and ESI (–), respectively; capillary temperature, 325°C; MS1 range, m/z 100–1,000; MS1 resolving power, 60,000 FWHM; number of data-dependent scans per cycle, 4; MS/MS resolving power, 15,000 FWHM; normalized collision energy, 30%; dynamic exclusion time, automatic.

### RNA-Seq and analysis.

Large intestinal ILC2s were sorted from WT mice and cultured in the presence of IL-2, IL-7, IL-25, and IL-33 and treated with or without DCA (100 μM) for 24 hours. ILC2s were lysed in RNA isolation reagent, and total RNA was isolated. Full-length cDNA was generated using a SMART-Seq HT kit (Takara); paired-end DNA libraries were generated and indexed using a Nextera XT Library Prep kit and then a Nextera XT Index kit. Barcoded samples were pooled and sequenced with the Illumina NovaSeq TM 6000 sequence platform, generating 2 × 150 bp paired-end reads. The reads were further filtered by Cutadapt (https://cutadapt.readthedocs.io/en/stable/, version: cutadapt-1.9). Filtered reads were mapped against the mm10 assembly of the Mus musculus genome (NCBI) using hisat2 (version 2.2.1). Significantly changed genes were identified by DESeq2. Significantly changed genes were used for pathway analysis with GSEA software.

### Bone marrow transfer.

To generate mixed bone marrow chimeras, bone marrow cells from *Gpbar1^−/−^* or *Nr1h4^−/−^* (CD45.2/CD45.2) mice were mixed at 1:1 ratio with WT (CD45.1/CD45.1) cells, and a 5 × 10^6^ cell mixture was i.v. injected into *Rag2^–/–^Il2rg^–/–^* mice irradiated at 700 rads. Chimeric mice were treated with antibiotics (sulfamethoxazole and trimethoprim; Hi-Tech Pharmacal) for 2 weeks after transplantation. After 4 weeks of hematopoietic reconstitution, mice received DCA treatment for 2 weeks and were analyzed 6 weeks after reconstitution.

### Insulin disulfide reduction assay.

The insulin disulfide reduction assay was performed as described previously with slight modifications. Briefly, aliquots of TMX2 (novo protein, HY-P71371) or purified recombinant human Trx was preincubated in a 90 μL reaction mixture (50 mM Tris-HCl, pH 7.4, 1 mM EDTA, 0.3 mM DTT) at room temperature for 15 minutes. The reaction was started by adding 10 μL of 10 mg/mL bovine insulin (Solarbio), and the change in the absorbance at 595 nm was recorded at room temperature. The nonenzymatic reduction of insulin by DTT was recorded as a control (56).

### Immunoblot analysis.

Cells were lysed in RIPA lysis buffer (50 mM Tris-HCl pH 7.4, 150 mM NaCl, 1 mM EDTA-2Na, 1% Triton X-100, 1% sodium deoxycholate, and 0.1% SDS) freshly supplemented with 1 × phosphatase inhibitor tablets and 1 × protease inhibitor cocktail tablets and phenylmethanesulfonylfluoride (1 mM). Equal amounts of protein were loaded and separated on 10% SDS-PAGE gel and transferred onto PVDF membranes (MilliporeSigma). The membranes were blocked with 5% skim milk in TBST for 1 hour at room temperature and incubated with indicated primary antibodies overnight at 4°C. After washing 3 times for 5 minutes with TBST, membranes were incubated with corresponding secondary HRP-conjugated antibody diluted in 5% skim milk for 1 hour at room temperature. Membranes were washed 3 times for 5 minutes with TBST, and then detected using the enhanced chemiluminescence reagents (Vazyme) and captured by a chemiluminescence imaging system (Tanon). Antibodies used in this study are listed in Supplemental Table 5.

### Misfolded protein quantification.

To quantify the relative abundance of misfolded protein aggregates in ILC2s, we utilized a Proteostat Aggresome detection kit (ENZ-51035-K100, Enzo Life Sciences). The Proteostat Aggresome detection assay was performed according to the manufacturer’s instructions. Briefly, cells seeded in 96-well plates were washed with PBS, fixed with 4% formaldehyde for 30 minutes at room temperature, permeabilized (0.5% Triton X-100, 3 mM EDTA) for 30 minutes on ice under gentle shaking, and stained with Proteostat dye (1:20,000 dilution) for 1 hour at room temperature. Nuclei were counterstained with DAPI. Samples stained with DAPI only served as a background control for Proteostat quantification. The cells were imaged with an Olympus FluoView FV3000 confocal microscope with excitation/emission (Proteostat) of 488/632 nm and DAPI (405/461 nm). Signal quantification was analyzed by ImageJ (NIH) software (57).

### Generation of released protein and ribosome-nascent chain complexes using a coupled rabbit reticulocyte lysate system.

Areg, IL-5, and IL-13 were synthesized in vitro using the TNT T7 Quick Coupled Transcription/Translation system (Promega) following the manufacturer’s instructions for 90 minutes at 30°C. For ER targeting experiments, released Areg, IL-5, and IL-13 with its native signal sequence were expressed in nuclease-treated rabbit reticulocyte lysate in the presence of canine pancreatic microsomes (Promega) following the manufacturer’s instructions. All samples were analyzed by SDS-PAGE or cytometric bead array (58, 59).

### Cytometric bead array.

Levels of Areg, IL-5, and IL-13 in colon tissue and rabbit reticulocyte lysate were measured by cytometric bead array analysis (Cloud-Clone Corp.) according to the manufacturer’s instructions.

### IP.

Cells were harvested and resuspended in 1 mL ice-cold IP buffer (50 mM Tris HCl, pH 7.4, 250 mM NaCl, 1% NP-40) containing protease inhibitor cocktail. Cells were lysed on ice for 30 minutes, and then centrifuged at 12,000*g* for 10 minutes at 4°C. Supernatant protein concentrations were measured using the BCA protein assay kit and normalized across samples. One-tenth of the volume of the lysate was set aside to be used as the input control. The remaining lysates were incubated with 10 μM biotin-labeled DCA or biotin and gently rotated at 4°C for 12 hours, and were then incubated with Pierce streptavidin magnetic beads at 4°C for 1 hour. After 3 washes with IP buffer, proteins bound to beads were eluted using loading buffer.

### Molecular docking.

The crystal structure of the TMX2 (PDB: 2DJ0) protein in complex with DCA was retrieved from the RCSB Protein Data Bank (https://www.rcsb.org/). The prediction of the active site of TMX2 protein was done by the PockDrug-Server server (http://pockdrug.rpbs.univ-paris-diderot.fr/cgi-bin/index.py?page=home). Molecular docking simulations were conducted using AutoDock Vina (version 1.5.7) after the addition of hydrogen atoms and removal of water molecules. Binding energies were calculated with AutoDock Vina to assess binding affinities, with values –6.7 kcal/mol indicative of strong interactions. Binding mode diagrams for TMX2-DCA complexes were generated using PyMOL.

### Surface plasmon resonance analysis.

Surface plasmon resonance analysis was run on a Biacore T200 system (Cytiva) with a Series S Sensor Chip CM5. Briefly, the ligand (TMX2 protein, novo protein, HY-P71371) was immobilized to the CM5 chip via covalent bonds to the amino acid residues in immobilization buffer. Subsequently, different concentrations of DCA were diluted in the analyte buffer and were injected into the flowing channel to allow interaction between TMX2 and DCA. The interacting phase included 120 seconds of association phase and 300 seconds of dissociation phase. The data were analyzed with Biacore T200 Evaluation software (Cytiva).

### Statistics.

All statistical analyses were performed using GraphPad Prism. Normality for all datasets was assessed using the Shapiro-Wilk test, and subsequent parametric or nonparametric tests were applied accordingly. Two-sided, 2-tailed unpaired *t* tests were employed for comparisons between 2 groups with a normal distribution; 2-sided Mann-Whitney *U* tests were used for comparisons between 2 groups with a non-normal distribution. For comparisons involving more than 2 groups, 1-way ANOVA was utilized with correction for multiple comparisons using Tukey’s test for parametrically distributed datasets and the Kruskal-Wallis test with Dunn’s correction for nonparametrically distributed datasets. The number of samples and experiments, as well as statistical tests used, are reported in each figure legend. Differences were considered significant at *P* values less than 0.05. Data are displayed as mean ± SD, unless otherwise indicated.

### Study approval.

All animal experiments were approved by the IACUC of Shandong University (ECSBMSSDU2020-2-057). All patients with UC were recruited from the Department of Gastroenterology, Qilu Hospital of Shandong University. This study was approved by the Clinical Ethical Committee of Shandong University (ECSBMSSDU2020-1-035).

### Data availability.

All data needed to evaluate the conclusions in the paper are present in the paper and/or the supplemental materials. Additional data related to this paper may be requested from the corresponding author. Values for all data points in graphs are reported in the Supporting Data Values file. The RNA-Seq data have been deposited in the NCBI’s Gene Expression Omnibus (GEO GSE294014).

## Author contributions

SL, YY, LG, and DY designed and supervised the research. QT and JS performed most of the experiments. HL, XG, XY, PH, YM, WX, and HM provided help with experiments. YC performed the bioinformatics analysis of the RNA-Seq data. MZ and YZ provided help with animal models. YL and SW carried out human sample collection and clinical data analysis. SL, QT, and JS wrote the manuscript. All authors read and provided feedback on the manuscript.

## Funding support

- National Key R&D Program of China (2020YFA0804400).
- National Natural Science Foundation of China (82321002, 82071854, 32422024, 82300615, 82270578, 82500657, 82502204).
- Taishan Scholars Program of Shandong Province (tsqn202306343).
- China Scholarship Council program (202306220313).
- Natural Science Foundation of Shandong province (ZR2022QH305).
- Basic Research Program of Jiangsu (BK20250421).

## Supplementary Material

Supplemental data

Unedited blot and gel images

Supporting data values

## Version 1 04/08/2026

Electronic publication
