# Supplementary material for: Vancomycin eliminates gut deoxycholic acid, restoring ER proteostasis in ILC2s and relieving colitis
Source: JCI Insight. 2026 Apr 8;11(7):e197470. doi: 10.1172/jci.insight.197470 (PMC13134711; doi:10.1172/jci.insight.197470)
Supplement: Supplemental data [file jciinsight-11-197470-s221.pdf]

Figure S1

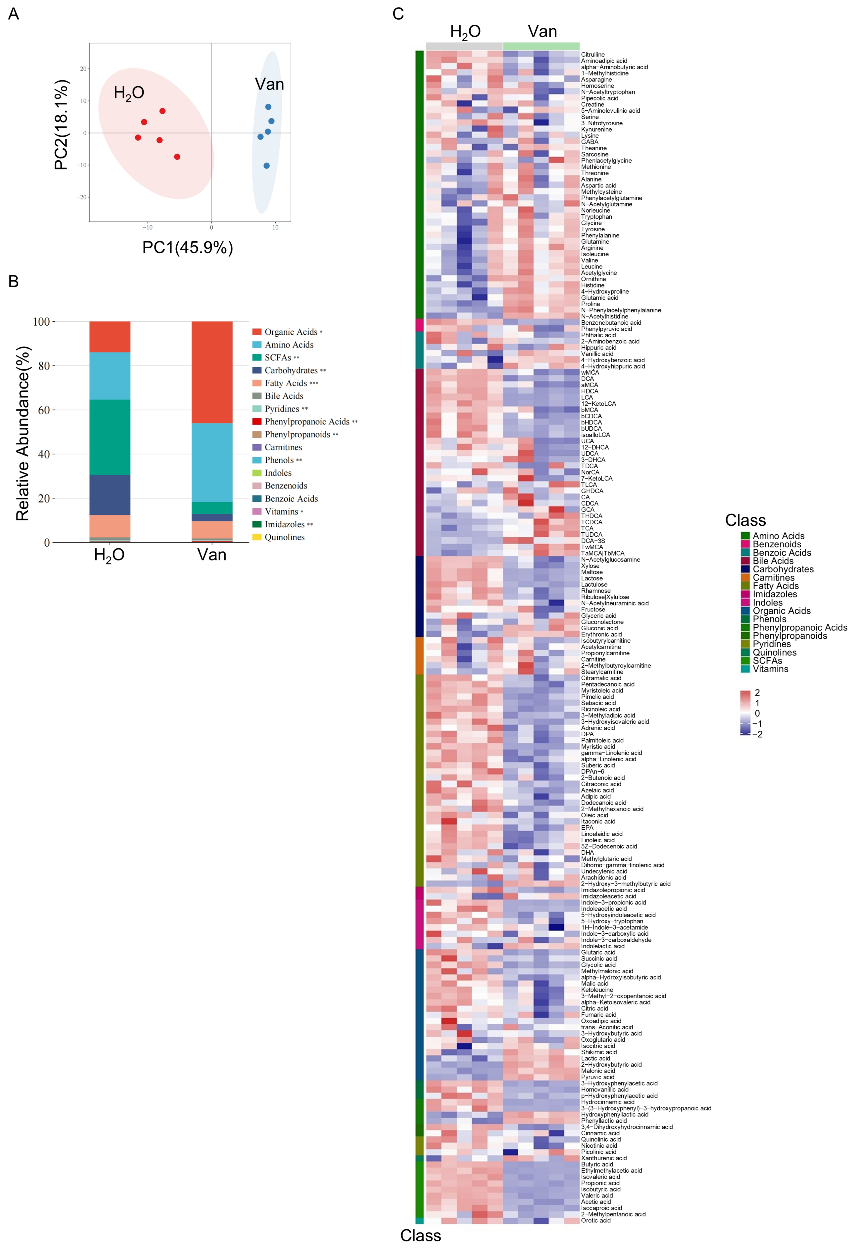

Supplementary Figure S1. Metabolite profiles in Vancomycin treated mice in feces

(**A–C**) WT Mice were gavaged a 5-day H_2O or Vancomycin after the 2.5% DSS administration for 5 days. (A) Principal coordinate analysis (PCA) score plot of fecal metabolite in separately housed mice treated or untreated with vancomycin. (B) The relative abundance of each metabolite classes between the H_2O group and vancomycin group. (C) Heat map of metabolite between the H_2O group and vancomycin group.

Figure S2

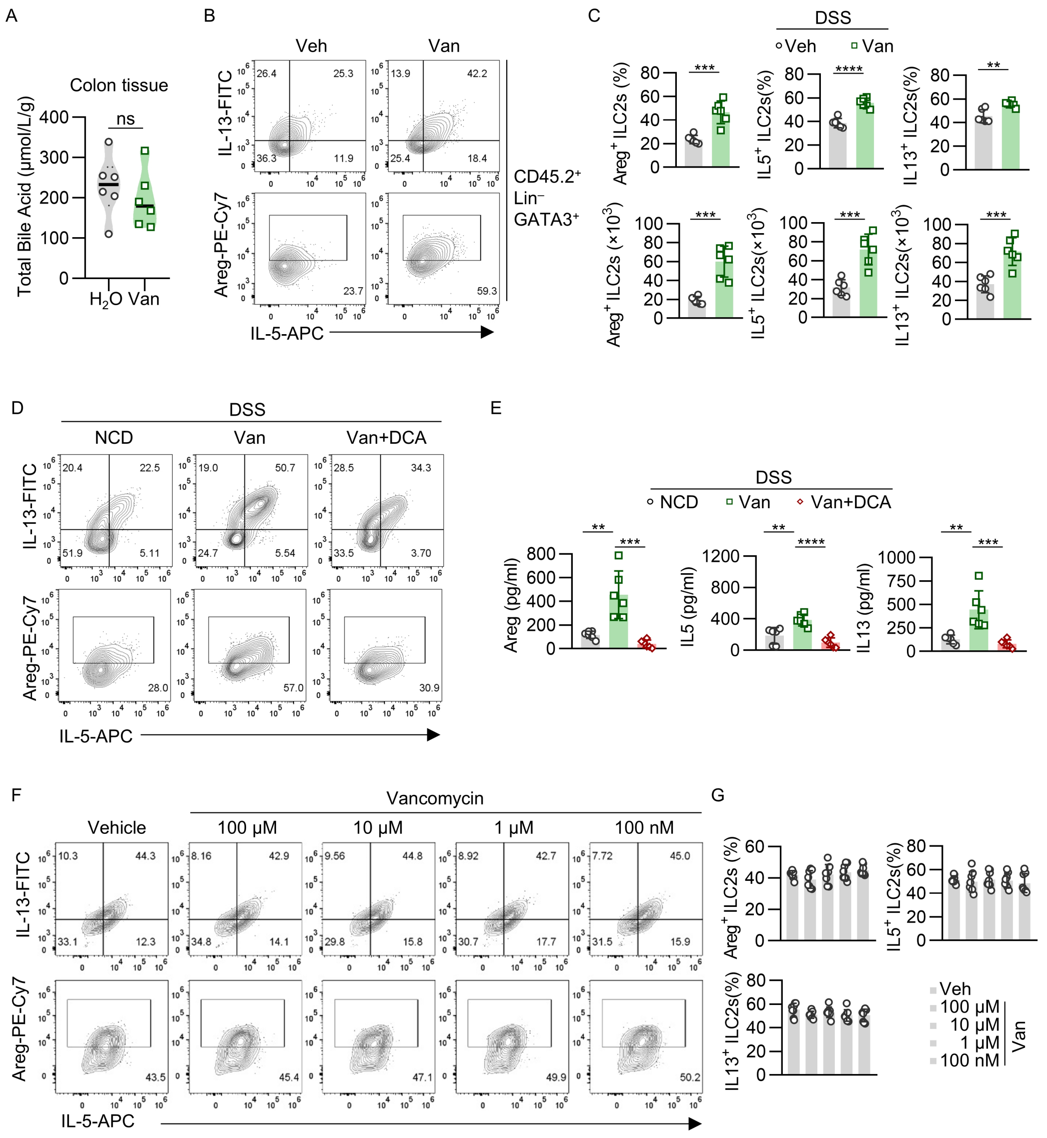

Supplementary Figure S2. Vancomycin promotes ILC2s indirectly

(**A**) The contents of total bile acid in colon tissue after Vancomycin gavage. n = 6 mice per group. The experiment was repeated three times.

(**B** and **C**) (B) Representative flow cytometry plots. (C, top) Percentages of Areg$^+$, IL-5$^+$, and IL-13$^+$ ILC2s (CD45.2$^+$Lin$^-$GATA3$^+$) and (C, bottom) absolute numbers of Areg$^+$ ILC2s, IL-5$^+$ ILC2s, and IL-13$^+$ ILC2s in large intestine. n = 6 mice per group. The experiment was repeated three times.

(**D** and **E**) WT mice were gavaged a 5-day H_2O or Vancomycin after the 2.5% DSS administration for 5 days. One group of Vancomycin-gavaged mice simultaneously

were fed a 2% DCA diet on day 5 to day 10. (D) Representative flow cytometry plots. (E) The concentrations of Areg, IL-5 and IL-13 in colonic tissue lysates were detected by Cytometric Bead Array. n = 6 mice per group. The experiment was repeated three times.

(**F** and **G**) Large intestinal ILC2s were sorted from WT mice and cultured in the presence of IL-2, IL-7, IL-25, and IL-33 for 5 days. ILC2s were treated with Vancomycin at the indicated concentrations for 24 h. (F) Representative flow cytometry plots. (G) Percentages of Areg$^+$, IL-5$^+$, and IL-13$^+$ ILC2s (CD45.2$^+$Lin$^-$GATA3$^+$) in large intestine. n = 8 wells per group. The experiment was repeated three times.

Data are represented as mean ± SD. The two-sided unpaired t test or two-sided Mann–Whitney U test was used in panel **A** and **C**, depending on data normality. One-way ANOVA with Tukey's multiple-comparison test or the Kruskal–Wallis test with Dunn's multiple-comparison test was used in panels **E** and **G**, based on data normality. The statistical methods and exact P values are provided in the Supporting Data Values file. Significance reported as $^{**}p < 0.01$, $^{***}p < 0.001$, $^{****}p < 0.0001$.

Figure S3

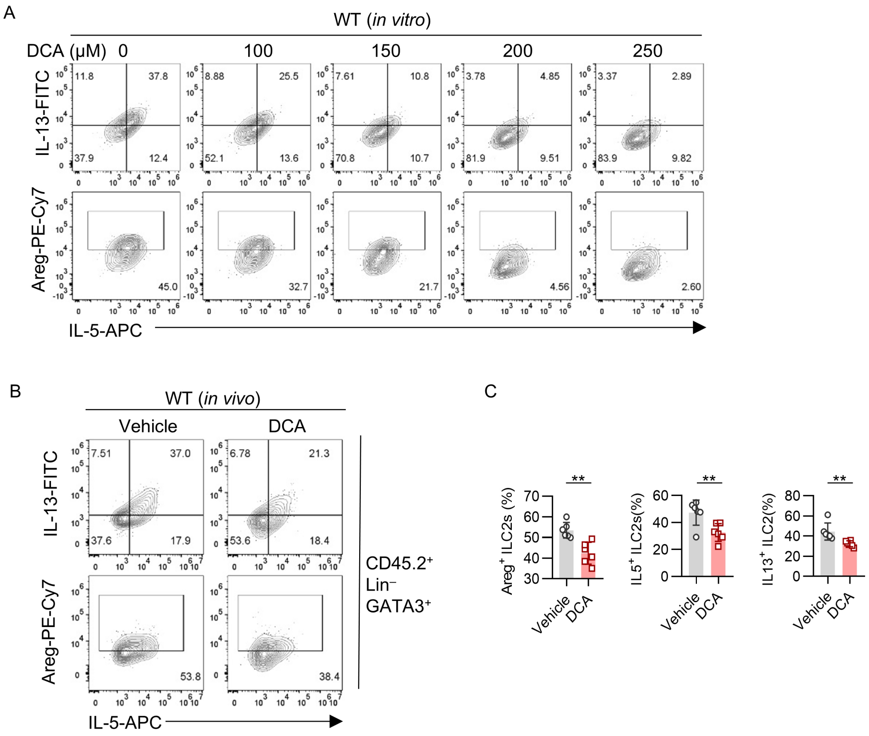

Supplementary Figure S3. DCA inhibits ILC2 function

(**A**) Representative flow cytometry plots. Large intestinal ILC2s were sorted from WT mice and cultured in the presence of IL-2, IL-7, IL-25, and IL-33 for 5 days. ILC2s were treated with DCA at the indicated concentrations for 24 h.

(**B** and **C**) WT mice were injected intraperitoneally (i.p.) with DCA or DMSO for 14 days. (B) Representative flow cytometry plots. (C) Percentages of Areg$^+$, IL-5$^+$, and IL-13$^+$ ILC2s (CD45.2$^+$Lin$^-$GATA3$^+$) in large intestine. n = 6 mice per group. The experiment was repeated three times.

Data are represented as mean ± SD. Statistical analysis was performed using unpaired two-tailed t test. Significance reported as **p < 0.01.

Figure S4

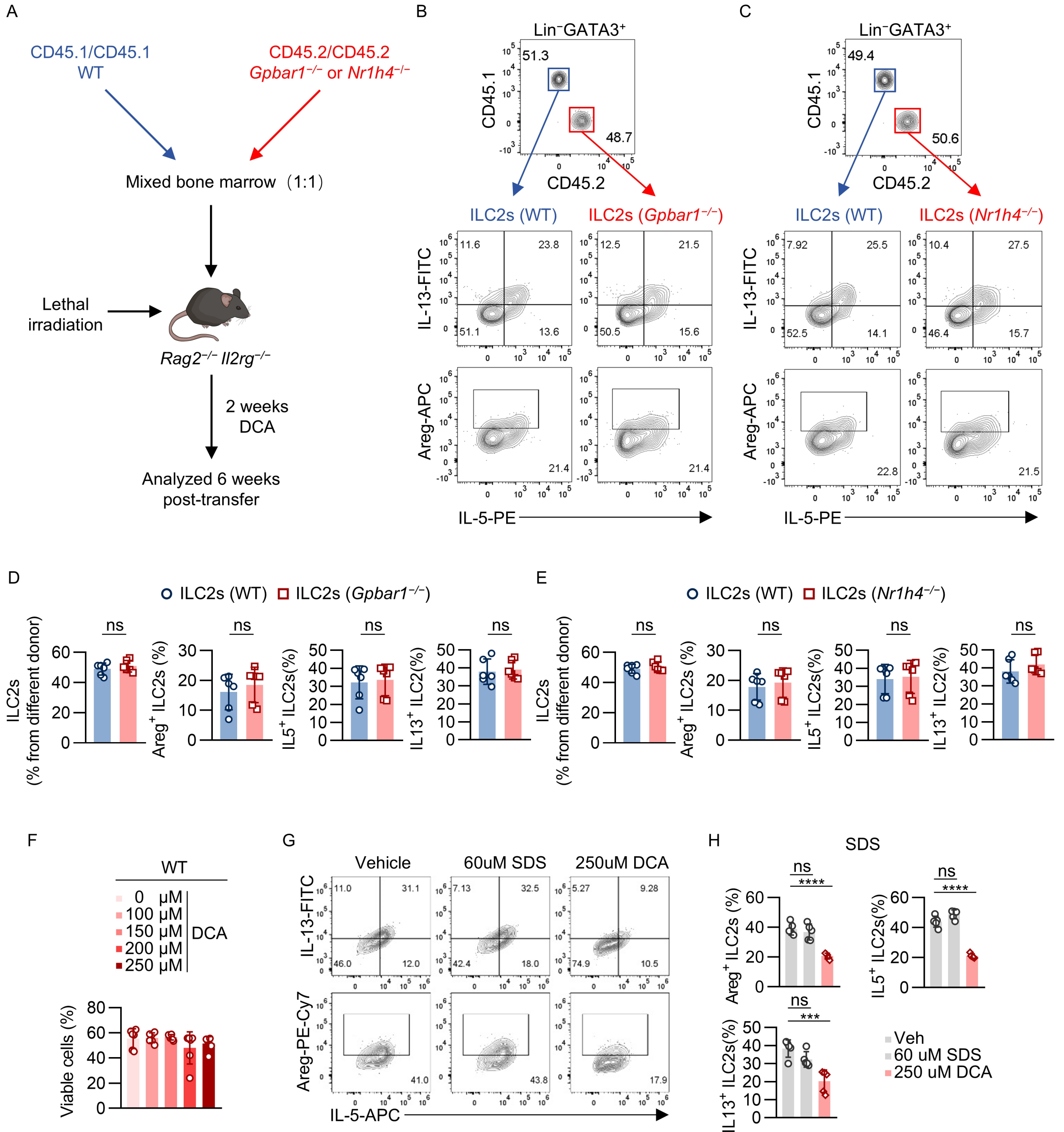

Supplementary Figure S4. DCA suppresses colonic ILC2 function independently of receptor signaling and without inducing cell death via detergent-like activity

(**A–E**) The lethally irradiated *Rag2*$^{-/-}$*Il2rg*$^{-/-}$ recipient mice were transferred with an equal mixture (1:1) of bone marrow cells from WT (CD45.1/CD45.1) and either *Gpbar1*$^{-/-}$ (CD45.2/CD45.2) or *Nr1h4*$^{-/-}$ (CD45.2/CD45.2) donor mice. (A) Experimental strategy. (B) Flow cytometry analyses of CD45.1 (WT ILC2s) and CD45.2 (*Gpbar1*$^{-/-}$ ILC2s) expression (upper), Areg, IL-5 and IL-13 expression (bottom) after gating on indicated populations. (C) Flow cytometry analyses of CD45.1 (WT ILC2s) and CD45.2 (*Nr1h4*$^{-/-}$ ILC2s) expression (upper), Areg, IL-5

and IL-13 expression (bottom) after gating on indicated populations. (D) Proportion of ILC2s (Lin^{-}GATA3^{+}) from different donors, and percentages of Areg^{+}, IL-5^{+}, and IL-13^{+} cells in recovered WT or *Gpbar1*$^{-/-}$ ILC2s. n = 6 mice per group. The experiment was repeated twice. (E) Proportion of ILC2s (Lin^{-}GATA3^{+}) from different donors, and percentages of Areg^{+}, IL-5^{+}, and IL-13^{+} cells in recovered WT or *Nr1h4*$^{-/-}$ ILC2s. n = 6 mice per group. The experiment was repeated twice.

(**F**) Large intestinal ILC2s were sorted from WT mice and cultured in the presence of IL-2, IL-7, IL-25, and IL-33 for 5 days. ILC2s were treated with DCA at the indicated concentrations for 24 h. Flow cytometry analysis of viability in ILC2s. n = 6 mice per group. The experiment was repeated three times.

(**G** and **H**) Large intestinal ILC2s were sorted from WT mice and cultured in the presence of IL-2, IL-7, IL-25, and IL-33 for 5 days. ILC2s were treated with SDS (60 μM) or DCA (250 μM) for 24 h. (E) Representative flow cytometry plots. (F) Percentages of Areg^{+}, IL-5^{+}, and IL-13^{+} ILC2s (CD45.2^{+}Lin^{-}GATA3^{+}). n = 5 wells per group. The experiment was repeated twice.

Data are represented as mean ± SD. The two-sided unpaired t test or two-sided Mann–Whitney U test was used in panel **D** and **E**, depending on data normality. One-way ANOVA with Tukey's multiple-comparison test or the Kruskal–Wallis test with Dunn's multiple-comparison test was used in panels **F** and **H**, based on data normality. The statistical methods and exact P values are provided in the Supporting Data Values file. Significance reported as ***$p < 0.001$, ****$p < 0.0001$.

Figure S5

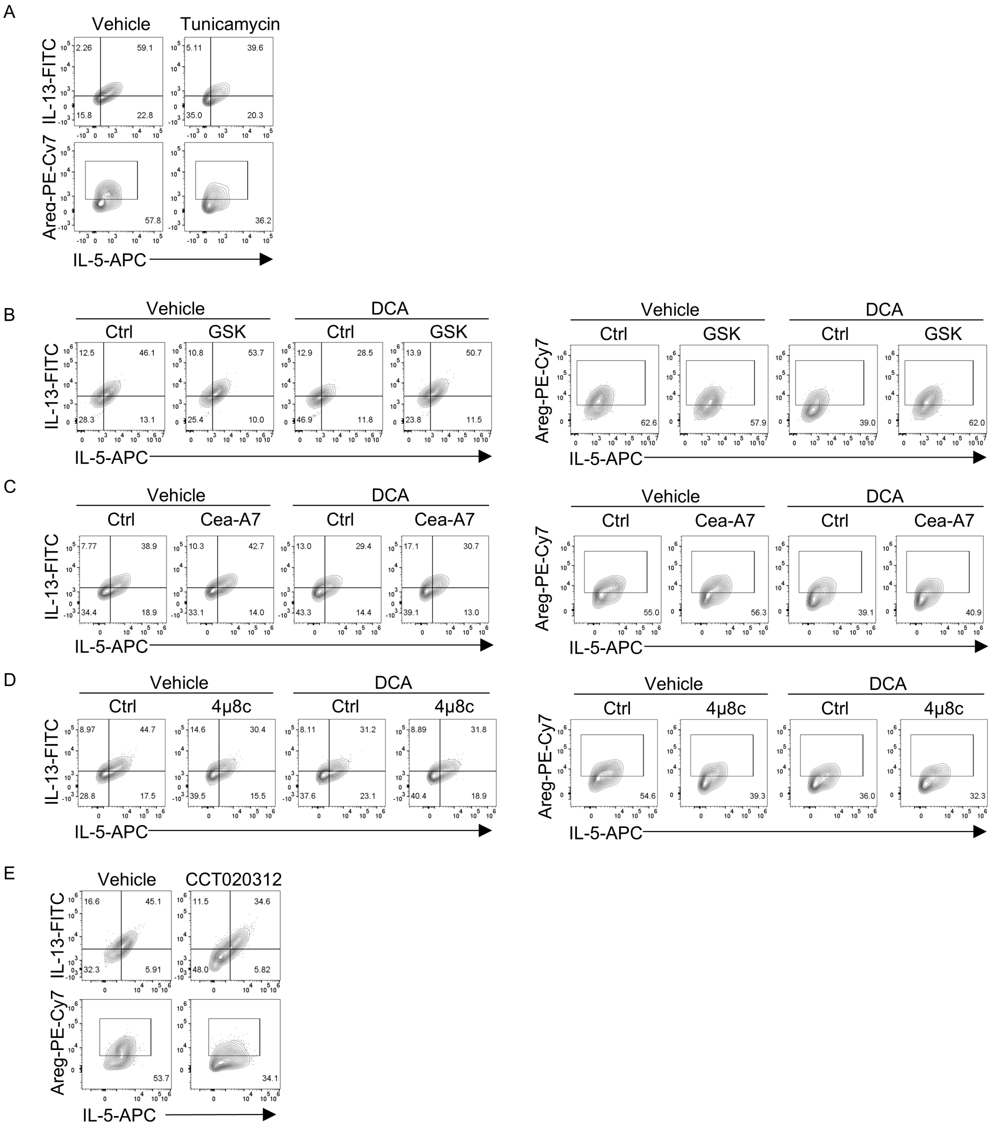

Supplementary Figure S5. DCA enhances UPR in ILC2s

(**A–E**) Representative flow cytometry plots. Large intestinal ILC2s were sorted from WT mice and cultured in the presence of IL-2, IL-7, IL-25, and IL-33 for 5 days. (A) ILC2s were treated with ER stress activator (tunicamycin, 0.3 μM) for 24 h. ILC2s were treated with DCA (100 μM) and ER stress inhibitors targeting (B) PERK phosphorylation (GSK, 10 μM), (C) ATF6 (Cea-A7, 10 μM), (D) IRE1α (4μ8c, 20 μM) and (E) PERK phosphorylation activator (CCT020312, 10 μM).

Figure S6

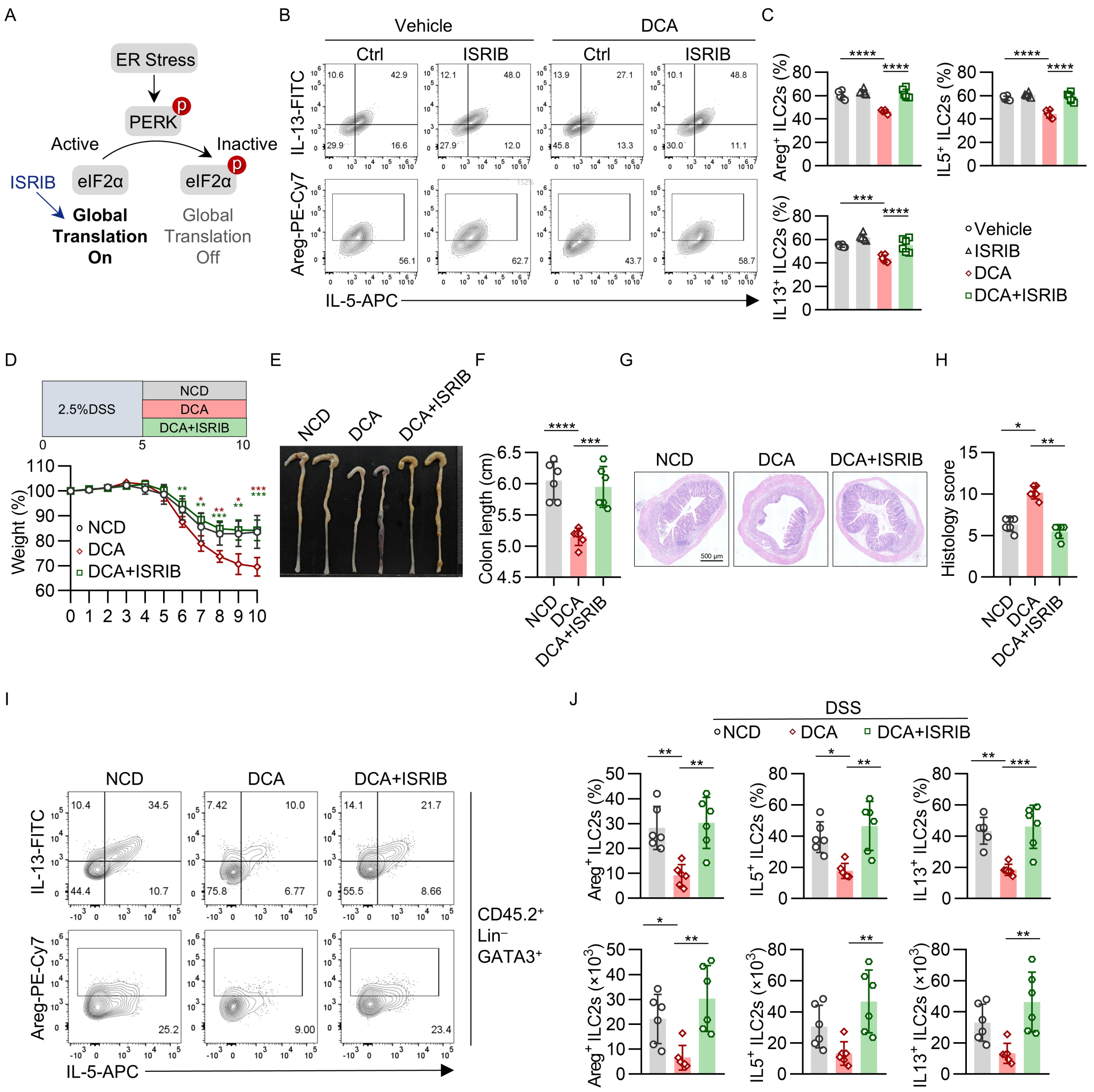

Supplementary Figure S6. PERK-eIF2a branch of UPR mediates the inhibitory effects of DCA on ILC2 function and colitis recovery

(**A–C**) Large intestinal ILC2s were sorted from WT mice and cultured in the presence of IL-2, IL-7, IL-25, and IL-33 for 5 days. ILC2s were treated with DCA (100 μM) and PERK phosphorylation inhibitor (ISRIB, 10 μM) for 24 h. (A) Schematic illustration of the PERK-eIF2α arm of the UPR. (B) Representative flow cytometry plots. (C) Percentages of $Areg^+$, $IL\text{-}5^+$, and $IL\text{-}13^+$ ILC2s ($CD45.2^+Lin^-GATA3^+$). n = 6 wells per group. The experiment was repeated three times.

(**D–J**) WT mice were fed a 5-day NCD or DCA after the 2.5% DSS administration for 5 days. One group of NCD-fed or DCA-fed mice simultaneously injected

intraperitoneally (i.p.) with ISRIB or DMSO for 5 days. (D, top) The experimental outline is shown. (D, bottom) Weight course. The red * represents the comparison between the NCD and DCA groups, the green * represents the comparison between the DCA and DCA+ISRIB groups. (E) Representative image of colons and (F) statistic data of colon length. (G) H&E-stained histological sections. Scale bar, 500 µm. (H) Histopathology scores. (I) Representative flow cytometry plots. (J, top) Percentages of Areg$^+$, IL-5$^+$, and IL-13$^+$ ILC2s (CD45.2$^+$Lin$^-$GATA3$^+$) and (J, bottom) absolute numbers of Areg$^+$ ILC2s, IL-5$^+$ ILC2s, and IL-13$^+$ ILC2s in large intestine. n = 6 mice per group. The experiment was repeated three times.

Data are represented as mean ± SD. One-way ANOVA with Tukey’s multiple-comparison test or the Kruskal–Wallis test with Dunn’s multiple-comparison test was used in panels **C**, **D**, **F**, **H**, and **J**, based on data normality. The statistical methods and exact P values are provided in the Supporting Data Values file. Significance reported as *$p < 0.05$, **$p < 0.01$, ***$p < 0.001$, ****$p < 0.0001$.

Supplemental table 1. Metabolite profiles in Vancomycin-treated compared to H_2O-treated in feces.

Class	Metabo	C1	C2	C3	C4	C5	W1	W2	W3	W4	W5	log2fc	p
Phenyl	Hydroc	100.8	21.8	38.0	109.2	49.6	0.2	0.2	0.5	0.4	0.3	-7.65	0.0218
Bile Ac	DCA	165.0	130.1	238.6	244.3	127.4	0.1	1.9	1.4	4.0	0.9	-6.78	0.0022
Phenol	3-Hydr	77.5	29.8	31.3	61.9	28.4	0.4	0.7	0.1	0.6	0.6	-6.55	0.0109
Bile Ac	12-Ket	34.8	12.5	63.5	24.2	20.3	0.3	0.5	0.3	0.6	0.4	-6.20	0.0259
SCFAs	Butyric	3317.4	1613.3	2226.9	2568.0	2755.3	36.1	33.0	41.4	41.8	39.0	-6.03	0.0010
Bile Ac	HDCA	27.0	8.7	22.4	22.6	7.4	0.2	0.9	0.1	0.3	0.1	-5.77	0.0123
Bile Ac	LCA	20.3	14.2	14.5	21.0	9.5	0.2	0.3	0.1	0.6	0.3	-5.70	0.0019
Bile Ac	wMCA	287.4	89.9	256.8	295.4	108.4	9.2	23.1	0.4	1.6	0.1	-4.92	0.0107
SCFAs	Ethylm	47.3	50.8	33.4	85.3	73.4	1.8	2.0	2.0	2.2	2.1	-4.85	0.0039
SCFAs	Propion	7356.3	4547.1	5735.4	8901.6	4146.7	227.1	248.4	211.9	169.4	225.3	-4.83	0.0026
SCFAs	Isovale	34.8	52.4	61.6	55.3	103.1	2.4	1.6	2.2	2.8	2.0	-4.80	0.0064
Phenyl	3-(3-H	3.1	2.1	1.0	4.6	1.9	0.1	0.1	0.1	0.1	0.1	-4.80	0.0164
Carboh	N-Acet	292.8	185.2	172.4	263.0	289.8	9.6	30.3	2.6	0.9	11.6	-4.45	0.0007
SCFAs	Isobuty	123.4	160.6	182.2	166.6	201.2	8.5	7.9	7.9	9.0	10.0	-4.27	0.0003
Bile Ac	bHDCA	8.4	1.7	5.8	4.6	2.1	0.2	0.6	0.2	0.1	0.3	-4.00	0.0262
Carboh	Maltos	2461.8	1408.2	2290.1	2748.7	561.6	130.1	136.7	106.8	133.4	155.0	-3.84	0.0118
Carboh	Lactos	2386.5	1358.4	2148.4	2634.1	571.1	135.8	144.1	98.2	137.1	167.1	-3.74	0.0112
Carboh	Xylose	9004.9	5017.3	5054.5	9330.1	6229.6	552.8	1137.2	231.3	303.7	543.0	-3.65	0.0021
SCFAs	Valeric	276.0	190.8	265.4	232.2	181.0	21.9	15.8	18.7	21.0	21.0	-3.54	0.0004
Indoles	Indole-	5.9	1.6	2.5	3.1	2.5	0.3	0.3	0.2	0.4	0.2	-3.52	0.0182
Bile Ac	aMCA	78.7	17.8	67.2	66.0	19.4	5.6	16.2	0.2	0.6	0.3	-3.44	0.0230
Bile Ac	isoallol	8.6	2.0	8.9	3.9	2.2	0.4	0.5	0.3	0.8	0.5	-3.36	0.0389
Organi	Glutari	54.4	47.9	19.4	25.9	23.7	3.0	4.0	2.4	4.6	4.4	-3.21	0.0123
Bile Ac	bCDCA	9.7	2.4	10.7	6.6	2.7	0.6	2.4	0.0	0.3	0.3	-3.16	0.0278
Carboh	Lactulo	51.2	41.8	21.4	40.4	17.8	5.7	4.8	5.1	4.1	5.1	-2.80	0.0098
Bile Ac	bUDCA	1.3	0.5	1.2	1.2	0.4	0.1	0.2	0.1	0.2	0.1	-2.78	0.0151
Phenol	Homov	9.7	7.1	2.4	8.1	4.3	1.0	1.0	1.0	0.8	0.7	-2.78	0.0145
Indoles	Indolea	1.3	1.7	4.8	5.4	2.5	0.4	0.5	0.5	0.6	0.5	-2.64	0.0329
Fatty A	Pentad	311.6	169.9	147.9	257.5	109.2	38.3	39.3	22.2	14.8	47.9	-2.62	0.0102
SCFAs	Acetic	17621.7	10536.8	18773.1	18621.2	15480.6	3193.8	3272.7	2309.0	2289.5	3444.1	-2.48	0.0008
Amino	Citrulli	1481.5	1523.8	1164.6	647.9	1196.6	155.3	228.9	148.5	276.6	342.1	-2.38	0.0027
SCFAs	Isocapr	2.5	1.9	3.1	4.0	2.5	0.7	0.4	0.6	0.6	0.5	-2.37	0.0029
Carboh	Rhamn	390.5	185.9	103.4	424.5	338.5	52.5	88.4	25.6	48.0	70.8	-2.34	0.0189
Fatty A	Citram	8.5	8.0	8.5	3.2	6.4	1.8	1.6	0.4	1.8	1.4	-2.30	0.0044
Imidaz	Imidaz	3.2	3.0	2.2	4.0	6.5	1.1	0.8	0.6	0.6	1.0	-2.26	0.0141
Fatty A	Myristo	10.6	8.7	7.4	8.1	10.8	1.9	2.0	1.4	1.9	2.9	-2.19	0.0002
Fatty A	Pimelic	6.4	4.3	2.9	7.3	4.0	0.9	0.7	0.8	1.4	1.7	-2.18	0.0076
Fatty A	Sebacic	11.6	8.4	9.2	14.8	8.2	2.5	2.1	1.9	2.5	3.0	-2.11	0.0028
Benzen	Benzen	1.9	1.7	1.2	1.5	1.3	0.3	0.4	0.4	0.3	0.4	-2.07	0.0004
Carboh	Ribulos	691.1	368.3	292.5	569.1	355.6	131.0	151.2	76.9	110.1	155.9	-1.86	0.0106
Fatty A	3-Meth	1.7	1.2	0.8	1.1	0.9	0.2	0.4	0.4	0.4	0.3	-1.80	0.0054
Bile Ac	bMCA	560.4	204.7	411.0	557.3	205.9	135.2	412.2	4.1	6.4	3.0	-1.79	0.0393
Organi	Glycoli	65.5	42.1	68.9	58.5	45.3	14.4	17.2	14.6	18.0	17.3	-1.78	0.0015
Organi	Succini	684.1	2384.3	293.8	780.6	199.9	249.7	269.1	165.9	306.0	294.2	-1.76	0.1964
Fatty A	Ricinol	5.5	6.1	5.7	5.7	3.3	1.5	1.1	1.3	1.5	2.5	-1.75	0.0008
Fatty A	Adreni	13.3	9.0	12.4	16.8	32.7	3.0	6.7	3.3	5.2	8.6	-1.65	0.0487
Fatty A	DPAn-	9.3	9.8	8.4	16.0	24.8	3.9	6.1	2.3	3.7	5.7	-1.65	0.0380
Fatty A	Citraco	0.3	0.1	0.3	0.1	0.2	0.1	0.1	0.1	0.0	0.1	-1.62	0.0421
Organi	Methyl	768.1	2031.0	336.6	1121.3	203.8	273.7	307.2	271.3	321.6	304.4	-1.59	0.1428
Fatty A	Adipic	7.5	9.2	4.4	7.4	5.4	2.8	2.8	0.7	2.1	3.6	-1.50	0.0037
Phenol	p-Hydr	17.8	56.4	51.0	17.3	36.2	12.7	14.6	10.6	13.0	13.4	-1.47	0.0480
Fatty A	3-Hydr	1.0	0.7	0.8	1.2	0.8	0.4	0.3	0.3	0.3	0.4	-1.47	0.0027
Fatty A	DPA	37.2	49.7	23.9	22.8	36.9	11.2	15.7	7.8	11.2	15.8	-1.47	0.0094
Fatty A	Palmito	189.3	291.9	169.1	168.0	342.8	74.1	109.5	46.7	63.4	125.7	-1.47	0.0110
Amino	Aspara	197.2	49.7	20.8	65.8	171.2	34.8	37.2	40.0	52.5	25.4	-1.41	0.1466
Fatty A	Myristi	99.7	70.1	72.8	85.0	69.3	30.5	30.6	28.7	28.6	34.6	-1.38	0.0009
Organi	alpha-I	2.9	2.3	3.9	2.7	2.4	1.3	0.9	0.8	1.0	1.5	-1.37	0.0020
Fatty A	alpha-I	382.3	603.3	427.1	485.5	284.9	114.6	104.6	151.6	206.4	277.7	-1.35	0.0042
Fatty A	gamma	30.9	51.1	36.6	41.3	36.9	11.0	15.0	11.7	16.8	22.9	-1.35	0.0006
Fatty A	Suberic	15.7	12.2	10.1	18.7	9.5	5.6	5.8	2.9	5.0	6.7	-1.34	0.0076
Amino	Amino	47.8	58.8	36.1	36.2	35.4	18.6	24.8	10.2	17.8	18.6	-1.25	0.0031
Carniti	Isobuty	0.4	1.0	0.1	0.6	1.1	0.3	0.5	0.2	0.2	0.2	-1.22	0.1092
Fatty A	Azelaic	74.6	52.9	39.0	49.3	32.8	25.6	22.7	17.6	18.4	22.7	-1.22	0.0155
Organi	Ketoleu	566.8	563.2	662.0	299.9	409.1	208.2	347.2	101.2	122.7	355.8	-1.14	0.0122
Organi	3-Meth	474.5	379.7	465.7	221.0	244.4	146.6	291.7	60.6	83.6	232.0	-1.13	0.0238
Carboh	N-Acet	371.5	337.7	144.8	285.8	470.1	166.6	211.4	99.4	36.8	231.5	-1.11	0.0316

Organi	Oxoadi	0.6	2.0	0.6	0.5	0.6	0.3	0.5	0.3	0.3	0.5	-1.11	0.1788
Vitami	Orotic	3.1	7.3	2.2	8.2	5.3	1.3	1.7	0.4	2.8	5.9	-1.10	0.1029
Phenyl	3,4-Dih	15.9	14.8	10.3	15.7	16.1	8.1	5.8	5.6	6.5	8.7	-1.07	0.0007
Amino	alpha-A	50.9	29.3	70.1	34.9	49.5	19.3	25.7	15.0	24.9	27.8	-1.06	0.0235
Pyridin	Quinol	4.3	4.7	1.9	4.1	2.3	2.7	1.8	0.8	1.6	1.5	-1.03	0.0315
Fatty A	2-Meth	0.3	0.4	0.2	0.5	0.5	0.2	0.2	0.2	0.2	0.2	-0.96	0.0213
Organi	alpha-K	211.7	224.4	265.7	108.1	125.1	94.1	158.5	33.8	57.7	147.0	-0.93	0.0528
Organi	Malic a	905.4	1258.8	661.6	667.0	880.9	352.6	752.9	218.7	367.8	609.9	-0.93	0.0219
Fatty A	Dodeca	50.3	29.8	34.3	65.9	50.6	34.0	25.6	19.4	18.4	26.3	-0.90	0.0255
Fatty A	Oleic a	2517.8	3259.5	2620.6	1459.1	2273.8	844.9	1450.9	841.1	1614.0	1840.5	-0.88	0.0165
Fatty A	2-Buten	34.1	71.5	46.9	61.9	58.0	24.2	50.7	18.2	21.4	38.2	-0.83	0.0272
Fatty A	EPA	54.6	102.5	60.1	80.5	79.1	33.1	45.5	30.5	36.6	70.8	-0.80	0.0216
Fatty A	Linolei	2809.3	3866.2	3169.9	3343.4	2944.4	1499.8	1443.4	1773.2	2065.3	2713.1	-0.76	0.0023
Organi	Citric a	1224.8	1416.7	750.2	864.9	935.1	681.7	697.2	440.4	598.2	638.8	-0.76	0.0219
Fatty A	5Z-Doc	0.7	0.8	0.7	0.8	0.5	0.2	0.3	0.4	0.5	0.7	-0.75	0.0391
Fatty A	Methyl	1.1	0.8	0.6	0.6	0.4	0.3	0.5	0.4	0.5	0.3	-0.75	0.0759
Pyridin	Nicotin	412.2	260.5	279.5	345.8	246.6	191.4	235.5	121.2	135.8	234.1	-0.75	0.0136
Fatty A	Linoela	114.9	165.3	128.6	147.5	117.4	64.6	62.9	75.1	85.6	112.8	-0.75	0.0033
Bile Ac	UCA	0.8	0.3	0.7	0.9	0.7	0.7	1.1	0.1	0.1	0.1	-0.73	0.2449
Indoles	5-Hydr	6.3	4.8	5.1	4.1	5.3	3.1	2.8	3.6	2.6	3.9	-0.67	0.0030
Benzoi	Phthali	4.1	3.2	1.6	3.3	1.9	2.4	2.3	1.3	1.4	1.6	-0.65	0.0954
Bile Ac	12-DH	1.2	0.5	2.5	1.2	0.5	1.0	2.3	0.1	0.2	0.2	-0.64	0.4753
Organi	3-Hydr	58.8	36.9	108.0	33.3	39.6	40.4	38.8	29.4	35.0	35.1	-0.63	0.2322
Phenyl	Cinnan	0.2	0.4	0.2	0.3	0.2	0.2	0.2	0.3	0.0	0.2	-0.61	0.2154
Fatty A	Itaconi	2.0	3.1	1.5	1.5	1.7	1.4	1.3	1.1	1.4	1.3	-0.60	0.0948
SCFAs	2-Meth	0.5	0.5	0.4	0.8	0.7	0.4	0.3	0.3	0.4	0.4	-0.59	0.0655
Fatty A	DHA	177.9	239.5	120.7	123.9	269.7	114.6	175.4	64.3	112.5	165.2	-0.56	0.1415
Indoles	1H-Ind	3.3	2.7	4.5	4.3	2.8	3.8	2.9	1.8	0.2	3.3	-0.55	0.1822
Indoles	5-Hydr	4.4	4.2	2.9	4.5	2.8	2.6	2.2	3.4	1.8	3.0	-0.52	0.0440
Indoles	Indole-	2.2	1.0	1.1	1.1	1.5	0.6	1.3	1.0	1.1	0.8	-0.52	0.1546
Amino	1-Meth	1.7	2.4	1.3	1.9	2.6	1.0	2.4	0.7	1.2	1.9	-0.47	0.1833
Bile Ac	NorCA	0.2	0.2	0.2	0.5	0.3	0.3	0.3	0.1	0.2	0.1	-0.46	0.2727
Fatty A	Undecy	0.4	0.3	0.5	0.8	0.4	0.3	0.5	0.2	0.4	0.3	-0.44	0.2503
Amino	Homos	105.9	47.4	31.3	48.3	110.5	47.3	91.3	24.8	31.5	58.0	-0.44	0.3985
Amino	N-Acet	2.5	1.3	1.9	2.1	2.4	2.3	2.4	0.8	0.7	1.4	-0.41	0.2685
Amino	3-Nitro	1.3	1.5	1.6	1.9	1.5	1.2	1.4	0.7	1.2	1.4	-0.40	0.0517
Bile Ac	TDCA	0.7	0.4	0.5	0.6	0.7	0.4	0.3	0.3	0.8	0.4	-0.35	0.2800
Fatty A	Dihom	121.8	133.0	58.2	48.5	148.7	59.4	170.0	29.7	55.8	93.8	-0.32	0.5403
Amino	Kynure	4.0	4.4	2.5	1.0	8.5	2.0	5.4	2.9	3.0	3.2	-0.30	0.5982
Organi	Fumari	104.2	225.5	162.7	98.8	189.4	96.1	231.6	30.8	63.3	219.0	-0.28	0.5777
Amino	Pipecol	7.3	5.6	5.9	2.9	6.1	4.7	4.8	2.9	5.0	5.4	-0.28	0.2758
Carboh	Fructos	206.2	96.3	93.8	95.7	107.0	126.2	222.8	33.6	33.0	78.1	-0.28	0.6285
Indoles	Indole-	1.7	1.3	2.1	1.0	1.0	1.0	2.1	0.5	0.8	1.5	-0.25	0.5433
Carniti	Acetylc	35.2	54.2	10.5	19.7	55.6	31.3	37.3	16.4	29.9	33.3	-0.24	0.5993
Benzoi	2-Amin	3.3	2.8	1.9	1.6	2.7	2.5	2.0	2.0	2.0	1.9	-0.23	0.3240
Benzoi	Hippur	0.2	0.3	0.2	0.4	0.6	0.2	0.1	0.2	0.6	0.4	-0.22	0.6870
Benzen	Phenyl	32.4	35.3	41.1	16.1	20.2	23.3	46.2	11.9	11.6	33.2	-0.20	0.6529
Amino	Lysine	2025.5	1665.1	1131.8	1480.4	2301.5	1296.5	1758.5	1226.5	1817.8	1405.1	-0.20	0.3880
Fatty A	Arachi	183.6	209.5	153.1	122.8	437.1	142.1	294.9	85.6	172.1	292.9	-0.16	0.7438
Amino	Serine	866.2	736.6	516.7	621.7	1123.1	607.8	1119.3	455.6	595.2	690.6	-0.16	0.6217
Amino	Creatin	781.5	1772.7	84.4	586.3	1503.5	976.4	1792.9	228.8	490.4	784.1	-0.15	0.8286
Bile Ac	UDCA	0.9	1.1	1.4	1.0	1.1	1.2	3.1	0.2	0.3	0.3	-0.13	0.8683
Carboh	Glyceri	73.9	94.2	145.4	58.1	69.2	68.5	92.0	48.0	68.7	125.7	-0.13	0.7202
Carniti	Propion	2.8	5.2	1.1	2.4	4.1	2.4	5.8	2.2	1.5	2.4	-0.11	0.8308
Carniti	Carniti	45.0	53.3	15.7	32.9	57.4	41.7	63.6	20.6	29.9	39.7	-0.06	0.8719
Amino	5-Amin	20.8	20.7	29.3	13.4	15.0	11.2	18.5	24.0	22.1	20.6	-0.04	0.8800
Bile Ac	7-Keto	0.6	0.5	0.5	0.4	0.5	0.5	1.0	0.3	0.3	0.4	-0.03	0.9438
Organi	trans-A	1.4	2.2	0.4	1.0	0.7	1.9	0.7	1.0	1.1	1.1	0.04	0.9275
Amino	Methio	438.8	329.2	202.7	276.5	553.0	297.4	552.0	230.0	391.7	416.7	0.07	0.8374
Amino	Phenla	0.2	0.2	0.1	0.2	0.2	0.2	0.1	0.2	0.3	0.2	0.08	0.6801
Pyridin	Picolin	7.6	8.5	10.3	7.4	8.0	3.9	8.0	9.5	13.2	9.9	0.08	0.7659
Bile Ac	TLCA	0.3	0.3	0.3	0.3	0.3	0.3	0.3	0.3	0.3	0.3	0.10	0.0732
Amino	GABA	29.5	35.9	17.9	18.1	18.5	27.0	35.8	14.4	22.4	30.5	0.12	0.7057
Organi	Oxoglu	150.6	171.9	341.6	54.4	70.4	187.9	287.1	61.0	62.9	259.6	0.12	0.8474
Amino	Sarcosi	38.3	30.7	26.9	28.9	43.9	34.6	51.6	21.9	34.4	42.3	0.13	0.5981
Carboh	Glucon	303.3	603.2	253.6	418.0	442.9	300.4	463.7	343.7	605.4	552.5	0.16	0.5781
Amino	Phenyl	0.3	0.2	0.3	0.4	0.4	0.5	0.3	0.3	0.3	0.4	0.17	0.4427
Amino	Asparti	1124.8	1294.1	909.6	930.1	1693.2	1338.5	1854.2	821.2	1045.9	1676.6	0.18	0.5321
Amino	Threon	402.2	297.7	122.0	247.8	556.7	338.8	618.8	208.0	316.4	367.7	0.19	0.6668

Bile Ac	GHDC	0.2	0.4	0.6	0.6	0.3	0.8	0.4	0.3	0.6	0.5	0.19	0.5828
Organi	Isocitri	22.5	24.9	12.8	22.4	20.3	24.4	26.0	22.6	18.8	27.2	0.21	0.2420
Amino	Alanin	2116.2	1511.7	1441.6	1561.2	2588.7	1922.0	3234.0	1194.5	1864.3	2667.0	0.24	0.4517
Amino	N-Acet	3.5	2.1	4.0	2.4	3.4	4.3	3.5	2.8	3.0	4.9	0.25	0.3043
Amino	Theani	1.0	1.9	1.2	0.3	0.9	0.4	1.6	1.7	1.3	1.3	0.25	0.5938
Amino	Trypto	89.3	91.4	56.3	74.8	124.1	94.1	149.8	64.8	100.6	111.8	0.26	0.3659
Benzoi	Vanilli	2.4	1.7	3.9	1.6	1.4	2.1	3.3	3.0	2.7	2.2	0.26	0.4396
Bile Ac	GCA	0.4	0.4	0.4	0.5	0.5	0.4	0.6	0.5	0.8	0.4	0.29	0.2867
Amino	Norleu	1.2	0.8	0.3	1.0	1.7	1.0	2.4	0.7	0.8	1.3	0.33	0.5445
Amino	Phenyl	502.9	382.9	201.2	350.5	606.7	447.3	761.3	364.9	516.8	612.6	0.40	0.2126
Amino	Tyrosi	382.5	300.9	178.3	279.5	530.8	379.8	632.5	320.8	373.1	504.7	0.40	0.2228
Carniti	2-Meth	0.2	0.4	0.1	0.2	0.4	0.3	0.7	0.1	0.2	0.3	0.40	0.4791
Amino	Glycin	975.3	1119.9	584.0	618.4	1583.8	1229.3	1732.2	917.4	1219.7	1481.2	0.43	0.1790
Amino	Methyl	0.6	0.3	0.2	0.3	0.9	0.6	0.9	0.5	0.5	0.7	0.44	0.3042
Amino	Ornithi	115.7	167.9	117.4	101.5	249.8	240.5	253.5	191.1	160.3	201.3	0.48	0.1111
Amino	Glutam	552.7	516.7	234.2	413.8	864.0	673.8	901.6	608.3	764.7	722.4	0.51	0.1073
Amino	Argini	972.9	869.7	618.7	948.9	1235.0	1076.7	1534.4	1183.2	1626.6	1283.3	0.53	0.0210
Imidaz	Imidaz	1.2	1.2	1.5	0.8	0.8	2.0	1.3	1.4	1.7	1.7	0.53	0.0277
Benzoi	4-Hydr	5.1	5.4	4.1	6.5	2.5	6.4	6.8	7.2	7.3	8.1	0.59	0.0183
Quinol	Xanthu	0.2	0.9	0.5	0.6	0.4	1.2	1.0	0.5	0.4	1.2	0.65	0.1757
Amino	Isoleuc	591.1	370.3	252.1	413.0	847.0	674.0	1111.0	544.1	729.7	857.3	0.66	0.0757
Amino	Leucin	947.3	627.0	357.4	677.8	1542.7	1160.3	1846.4	925.1	1297.0	1442.3	0.68	0.0843
Bile Ac	THDC	0.6	0.4	0.5	0.6	0.7	0.7	0.5	1.0	1.4	1.2	0.72	0.0804
Amino	Valine	602.9	419.2	288.4	397.8	934.7	778.7	1302.4	602.8	775.2	1035.3	0.77	0.0573
Amino	Acetylg	56.9	41.0	29.4	40.4	87.9	77.2	129.0	58.6	74.4	99.1	0.78	0.0514
Benzoi	4-Hydr	4.0	3.0	5.6	6.2	2.4	9.5	6.7	5.1	8.3	7.2	0.79	0.0177
Carniti	Stearyl	0.3	0.6	0.4	0.3	0.7	0.8	1.5	0.3	0.6	1.0	0.86	0.1537
Bile Ac	CDCA	1.9	1.7	3.1	1.6	2.4	3.7	9.5	2.0	2.2	1.9	0.86	0.2997
Organi	Shikim	9.4	3.3	3.7	5.5	5.1	12.2	10.2	8.9	11.0	7.0	0.87	0.0139
Carboh	Glucon	19.8	32.7	14.3	20.7	18.2	41.8	43.1	21.1	55.5	37.0	0.91	0.0255
Amino	Histidi	279.4	277.8	137.3	211.4	408.2	443.9	670.3	320.3	480.9	597.7	0.94	0.0147
Amino	Glutam	639.8	586.5	621.5	245.7	881.1	1479.1	1593.6	1017.6	1195.7	1343.0	1.16	0.0010
Bile Ac	3-DHC	4.6	2.6	3.3	3.8	2.8	16.8	21.6	0.9	1.3	1.3	1.30	0.3301
Amino	N-Pher	0.3	0.2	0.2	0.2	0.2	0.6	0.6	0.3	0.5	0.4	1.32	0.0038
Amino	4-Hydr	7.2	10.1	7.1	3.8	8.1	23.8	25.6	10.4	14.0	20.7	1.37	0.0132
Carboh	Erythr	2.3	6.1	2.5	1.9	3.5	10.0	8.4	8.4	8.5	12.0	1.54	0.0003
Amino	Proline	488.2	395.3	281.1	333.1	657.1	1656.2	1863.9	1016.7	1263.5	1344.3	1.73	0.0012
Indoles	Indolel	7.4	11.1	28.7	4.9	1.4	49.7	38.1	23.6	30.8	40.1	1.77	0.0042
Bile Ac	TCDC	0.5	0.4	0.4	0.4	0.6	0.8	1.1	3.5	1.5	2.1	1.92	0.0498
Phenyl	Hydrox	12.0	8.2	17.9	23.3	9.5	97.1	113.0	50.6	79.8	75.9	2.56	0.0020
Bile Ac	TCA	9.7	4.0	8.2	11.6	14.9	21.8	18.5	146.3	71.1	72.4	2.77	0.0714
Bile Ac	CA	5.8	4.3	11.2	6.1	9.1	56.9	183.2	2.6	5.4	2.9	2.78	0.2858
Amino	N-Acet	1.3	1.5	5.1	2.7	1.5	22.3	24.1	13.4	14.7	25.1	3.04	0.0013
Organi	Lactic	1152.5	3674.9	2268.4	693.0	3371.6	20858.9	15576.9	14066.0	29284.4	14117.0	3.07	0.0040
Bile Ac	TaMC	9.6	4.8	6.4	12.1	17.8	23.5	37.7	227.3	69.8	89.7	3.14	0.0940
Phenyl	Phenyl	8.7	12.5	26.4	4.0	4.7	109.0	107.7	82.0	118.7	84.0	3.15	3.07E-05
Bile Ac	TUDC	0.2	0.1	0.1	0.2	0.3	0.8	0.6	3.2	1.1	2.2	3.22	0.0449
Bile Ac	TwMC	1.0	0.6	0.6	1.2	1.5	3.8	1.7	17.9	8.7	14.2	3.24	0.0531
Organi	2-Hydr	19.1	12.2	14.7	12.2	11.8	180.4	146.0	77.7	170.1	98.0	3.26	0.0038
Fatty A	2-Hydr	14.1	13.8	16.6	11.3	7.1	205.4	152.6	110.7	216.2	150.6	3.73	0.0013
Organi	Maloni	16.7	17.2	8.2	12.1	12.8	246.3	111.5	203.5	224.5	233.1	3.93	0.0013
Organi	Pyruvi	14.8	15.6	24.1	9.5	14.5	261.6	382.0	119.5	267.7	364.3	4.15	0.0048
Bile Ac	DCA-3	0.3	0.9	0.5	0.2	0.1	71.6	112.3	1.8	2.0	7.0	6.67	0.1634

Supplemental table 2. Clinical characteristics and demographic information of UC participants.

**Patients with UC**	**1**		**2**		**3**		**4**	
**Age(years)**	19		63		21		72	
**Gender**	Male		Female		Female		Male	
**Weight(kg)**	64		72		52		55	
**BMI(kg/m^2)**	19.1		24.9		18.9		19	
**Vancomycin treatment**	Before	After	Before	After	Before	After	Before	After
**Truelove-Witts score**	Severe	Mild	Moderate	Mild	Severe	Mild	Severe	Moderate
**Bloody stools/d**	≥10	0	6-8	0	6-10	0	≥10	0
**Pulse (≥90 bmp)**	95	73	84	64	100	68	95	75
**Temperature(℃)**	36.5	36.4	36.8	36.5	36.2	36	39	36.1
**Hemoglobin(g/L)**	82	83	143	129	111	135	115	108
**ESR (≥30 mm/h)**	109	56	31	3	47	6	2	72
**CRP (0-10 mg/L)**	41.72	1.19	14.33	0.64	163.91	0.2	10.22	7.81

Supplemental table 3. Demographic information of healthy donors.

ID	Age (years)	Gender	Weight (kg)	BMI (kg/m^2)	Index
HC-1	55	Male	66.0	22.8	Colonic LPMNCs, Flow cytometry, DMSO treatment
HC-2	64	Female	68.0	25.0	Colonic LPMNCs, Flow cytometry, DMSO treatment
HC-3	55	Female	70.0	24.8	Colonic LPMNCs, Flow cytometry, DMSO treatment
HC-4	53	Male	80.0	27.7	Colonic LPMNCs, Flow cytometry, DMSO treatment
HC-5	60	Male	70.0	23.4	Colonic LPMNCs, Flow cytometry, DMSO treatment
HC-6	57	Male	86.0	28.7	Colonic LPMNCs, Flow cytometry, DMSO treatment
HC-7	20	Female	76.0	25.7	Colonic LPMNCs, Flow cytometry, DMSO treatment
HC-8	62	Female	70.0	23.9	Colonic LPMNCs, Flow cytometry, DMSO treatment
HC-9	56	Female	55.0	19.5	Colonic LPMNCs, Flow cytometry, DMSO treatment
HC-10	35	Male	85.0	27.8	Colonic LPMNCs, Flow cytometry, DMSO treatment
HC-11	36	Female	72.0	28.1	Colonic LPMNCs, Flow cytometry, GSK2606414 treatment
HC-12	28	Male	87.5	29.9	Colonic LPMNCs, Flow cytometry, GSK2606414 treatment
HC-13	68	Female	80.0	28.7	Colonic LPMNCs, Flow cytometry, GSK2606414 treatment
HC-14	58	Male	77.0	26.6	Colonic LPMNCs, Flow cytometry, GSK2606414 treatment
HC-15	63	Male	75.0	25.4	Colonic LPMNCs, Flow cytometry, GSK2606414 treatment
HC-16	67	Female	64.0	23.5	Colonic LPMNCs, Flow cytometry, GSK2606414 treatment
HC-17	61	Male	71.0	23.2	Colonic LPMNCs, Flow cytometry, GSK2606414 treatment
HC-18	60	Female	67.7	24.9	Colonic LPMNCs, Flow cytometry, GSK2606414 treatment
HC-19	68	Male	65.0	21.7	Colonic LPMNCs, Flow cytometry, GSK2606414 treatment
HC-20	46	Male	75.0	26.6	Colonic LPMNCs, Flow cytometry, GSK2606414 treatment
HC-21	36	Female	72.0	28.1	Colonic LPMNCs, Flow cytometry, DCA treatment
HC-22	49	Female	61.0	23.0	Colonic LPMNCs, Flow cytometry, DCA treatment
HC-23	50	Male	73.0	23.0	Colonic LPMNCs, Flow cytometry, DCA treatment
HC-24	50	Female	64.0	24.4	Colonic LPMNCs, Flow cytometry, DCA treatment
HC-25	63	Female	51.0	19.9	Colonic LPMNCs, Flow cytometry, DCA treatment
HC-26	35	Male	79.0	25.5	Colonic LPMNCs, Flow cytometry, DCA treatment
HC-27	43	Female	62.0	24.8	Colonic LPMNCs, Flow cytometry, DCA treatment
HC-28	36	Female	65.0	26.0	Colonic LPMNCs, Flow cytometry, DCA treatment
HC-29	38	Female	60.0	22.9	Colonic LPMNCs, Flow cytometry, DCA treatment
HC-30	56	Male	80.0	26.4	Colonic LPMNCs, Flow cytometry, DCA treatment
HC-31	63	Female	70.0	24.2	Colonic LPMNCs, Flow cytometry, DCA + GSK2606414 treatment
HC-32	60	Female	65.0	25.1	Colonic LPMNCs, Flow cytometry, DCA + GSK2606414 treatment
HC-33	65	Female	65.0	25.4	Colonic LPMNCs, Flow cytometry, DCA + GSK2606414 treatment
HC-34	75	Male	76.5	23.6	Colonic LPMNCs, Flow cytometry, DCA + GSK2606414 treatment
HC-35	56	Male	95.0	32.1	Colonic LPMNCs, Flow cytometry, DCA + GSK2606414 treatment
HC-36	57	Female	48.0	19.2	Colonic LPMNCs, Flow cytometry, DCA + GSK2606414 treatment
HC-37	75	Male	69.0	23.1	Colonic LPMNCs, Flow cytometry, DCA + GSK2606414 treatment
HC-38	49	Female	55.5	21.1	Colonic LPMNCs, Flow cytometry, DCA + GSK2606414 treatment
HC-39	55	Male	67.0	23.7	Colonic LPMNCs, Flow cytometry, DCA + GSK2606414 treatment
HC-40	63	Male	61.5	21.5	Colonic LPMNCs, Flow cytometry, DCA + GSK2606414 treatment

Supplemental table 4. Proteins Bound to DCA.

Actg2
Actc1
Acta1
Acta2
Hbb-b2
Hbbt2
3000002C10Rik
Rps9
Pcx
Pc
Dars1
Dars
Trappc11
Nefh
Dop1b
Vps13a
Naa25
Chd9
Atl3
Haus8
Myo7b
Slfn1
OTTMUSWSBG00059304
Sltm
Cabin1
Cracdl
Kif13b
Scn10a
H1-4
Fbxl18
Gm1141
Phip
2210010C04Rik
Dnah2
RP23-56I20.8-001
Ccdc25
Arid5b
Taf1d
L3mbtl1
Szt2
mKIAA0467
Shroom2
Shroom3
Ubr4
Atp2c2
Prr19
H2az2
H2az1
H2ax
H2ac21
Parp4
Apob
Ell
Hyal2
Gtf2h4
Bard1

Clec11a
Mkln1
Mos
Ucp1
Npr1
Npr-a
Hoxb9
Hoxc10
Hoxa10
Hoxa11
Hoxd9
Hoxd11
Hoxd10
Hoxa9
Hoxc9
Wnt2
Lmnb2
Lmna
Zfp42
Mboat4
Rcc2
Twist1
Fbl
Rpl29
Pkm
Osm
Atp5f1b
Kcnj3
Kcnj1
Renbp
Rpl19
Smad4
Rbbp6
Col17a1
Fbln1
Ak8
Igfn1
Treml4
Vrtn
Trim43a
Trim43c
Trim43b
Gm15737
Ckap2
Fbxo31
Ogfod1
Ccdc177
Gpr35
D5Ertd579e
Kiaa0232
Dipk2a
Otol1
Syce1l
Ermn
Cep44
Heatr5a
Trpc6
Adam12

Cnga2
Pptc7
Ift122
Rrp12
Osgepl1
Jade2
Hexim2
Gimap8
Scoc
Kif27
Hook2
Trpm4
Lrrc75a
Cep120
Dpp8
Arhgap33
Ankrd45
Tmco3
Gabra5
Txlng
Cdkn2aip
Frmd4a
Zc2hc1a
Zcchc3
Zdhhc14
Cep55
Lcmt2
Ttc39b
Tiparp
Scara3
Amer2
Phf20l1
Ccdc87
Akr7a2
Lonp1
Pum1
Mical3
Asb2
Tnfaip8l1
Sdha
Shtn1
Ahsa2
Acat1
Angptl8
Stx19
C1qtnf12
Top1mt
Rttn
Abcc9
Apbb1ip
Ndc1
Ttc29
Cant1
Cdhr5
Cox4i2
Hpx
Cfap44
Pip4k2c

Uncharacterized protein C6orf132 homolog
Tspan2
Tssk6
Homer3
Znhit2
Mbip
Erc1
Mrpl36
Ankrd17
Znf318
Ndufa3
Ddx28
Prkrip1
Narf
Mettl18
Zbed3
Chaf1b
Synj2
Ttyh1
Cers5
Btbd17
Mettl7b
Tsc1
Pik3ap1
Oga
Invs
Ddx24
Cpb2
Utp3
Pard6g
Tnfrsf19
Cdc42ep4
Dnajb1
Ppie
Cdyl
Cfap206
Pole
Heph
Tulp1
Elapor1
Ddr2
Pcdh9
Bcar3
Srrt
Spata1
Crybg1
Anapc5
Scn8a
Golga1
Cmas
Recql4
Lingo4
Gtf3c1
Spink5
Pigq
Fcer2a
Fcer2
Jakmip1

Casc1
Dnai7
Fubp1
Las1
Sst
Gm10775
Taldo1
Rab11fip1
Mast3
Gm11639
Ric8b
Pam16l
Spata19
Cyp27a1
Ccdc7a
Gtf3c6
Slc16a10
Tbc1d5
Adam6a
Adam6b
Gpr19
Pcnt
Gopc
Kidins220
Zfp410
Catsperb
Eml5
Lrguk
Fam135b
ARHGAP28
Lztr1
Vmn2r111
Vmn2r116
Sil1
Matr3
Cspp1
Faf2
Prr33
Dnah7b
Mpz
Pakap
Akap2
1810058I24Rik
Stmp1
4930433I11Rik
Ccdc50
Gsdmc4
Gsdmc
Braf
If1ia2
Ifnl3
Otud5
Zfp937
Zfp442
3300002I08Rik
Ndufaf7
Tnrc6c
Pcdhga6

Arl6
Epc1
Ankrd35
Hars
Hars1
Carf
Gm49340
Spata22
Clspn
Wdpcp
Gale
Vwa5b1
Lpcat2b
Asxl2
Clec3a
Cfap77
Hal
Ibtk
Kirrel2
Cdr2l
Wfdc1
Dlgap4
Pde4dip
Sptan1
Eprs
Eprs1
Zfp174
Herc2
Cacna1c
Cnnm4
Smtn
Stk16
Ddx23
Tmx2
Stxbp5
Ica1
Pikfyve
Tbc1d14
Il12rb2
Rps15a
Fbxo22
Selenbp1
Selenbp2
Gjb6
Kctd18
Etv1
Etv5
Etv4
Kcnma1
Myo18b
Krcc1
Cftr
Myo9b
Fcf1
Rnf25
Dennd4c
Cep192
Hk2

Col6a6
Lrrc7
Rgl1
Pnpla8
Arhgap17
Tjp3
Mdc1
Nol8
Hjurp
Cenpe
Mga
Taf12
Trpm2
Ndufa12
Pla2g6
Srsf11
Npepl1
Mtmr1
Fgfr3
Mlxip
Ppp1r35
Bcl3
Lipi
Fam98c
Mgat4b
Nrap
Mpdz
Pdia6
Bdp1
Bcorl1
Pkn2
Aoc2
Scart2
Xlr3c
Xlr3a
Scart1
Slitrk2
Themis2
Xlr3b
Xlr3
Slc43a1
Cobl
Ermp1
Lhx2
Nptn
Ip6k1
Senp6
Acadl
Gbf1
Wdr90
Samd11
Pfn1
Ccdc180
Usp13
Wfikkn2
Pramel45
Gm17078
Mta1

Mta3
SKI
Skiv2l
Por
Necap1
Snx21
Ncf2
Ccr8
Crlf1
Il1f10
Vmn2r112
Lmnb1
F830208F22Rik
Arhgap6
Appl2
Mob3b
Emilin2
Phyh
Hexa
Rps6ka1
Phaf1
Rad23b
Ptpn13
B3gnt2
Gm10642
Pxk
Unc5b
Serpina3h
Serpina3g
Serpina3n
Zfp384
Etv3
Sidt2
Snx13
Hspa5
Ccl9
Arpc5
Gbp2
Etl4
Skt
Morn1
Nipsnap1
Mill1
Siglech
Reln
Acad8
Clpp
Sf1
Ldha
Hs3st6
Ndufs1
Maoa
Gart
Pcdhb6
Kctd17
B3gnt3
Cyp2j13
Alg8

Mef2d
Gm10629
Nptx1
A130057D12Rik
Gm10631
Eftud2
Gm906
Gm8765
Tcfl5
1700001L05Rik
Or6c69
Chek1
Huwe1
Ets1
Ets2
Chek2
Fgl2
Otor
Uhrf1bp1
N4bp1
Spata33
Rpl15
Gm6525
Gm12258
Foxo6
Haus4
Ccdc92b
Hmgn2
NMDA2C
mKIAA1769
Helz2
Exoc4
Vps13d
Zfp975
Zfp976
Dhx37
Stat2
Kat2a
Kat2b
Hdac5
Notch2
Tha1
Cpne1
Gm28036
Rbm12
Rap250
Ncoa6
Arpc4
Mast1
Or52r1c
trypsinogen
Gm2663
1810009J06Rik
Mcm3ap
Tbc1d22b
Asz1
Myh1
Myh4

Ror1
Cntnap4
Copb1
Penk
Rusc2
Synm
5730596B20Rik
Sox6
Epb41l5
9530077C05Rik
Matcap2
Incenp
Dpp6
Cacnb3
Vangl2
Extl3
Agap3
Dsel
H1f0
H1-0
Epha3
B230217C12Rik
Dnaaf6b
Nfib
Ptpn14
Klhl1
Thoc7
Chchd2l
1700084P21Rik
1500015L24Rik
Map1b
Plekha5
Aldh1l1
Aldh1l2
Nos1
Psmc5
Clcn1
Oas1b
Oas1d
Oas1e
Oas1f
BC022960
Slc25a24
Vmn1r18
Ighg2b
Ppp1r37
Wdr74
Ddx20
Hmmr
Or1ab2
Or8k41
Olfr1285
Or4k37
Or4k38
Or4k40
Or4c127
Or4c52
Or4c126

pol
Itga9
Gzmn
AY036118
Slc38a10
Mrps23
Hmgb2
Ccdc178
Tubb4b
Tubb3
Tubb5
Tubb4a
Gramd3
Slxl1
Gm16405
Gm14625
4922502B01Rik
Tab3
Il17ra
Gpa33
1700063H04Rik
Rad51d
Rad5113
TRAD/RAD51L3
Elp1
Klra21
Klra8
Zim1
Ccdc154
Atp13a4
Rbm46
Arhgef40
Arfgap1
Gsdma3
Atad5
Disc1

Supplemental table 5. Reagent or resource.

REAGENT or RESOURCE	SOURCE	IDENTIFIER
Antibodies		
Anti-mouse CD3e, APC-eFluor 780	eBioscience	Cat# 47-0031-82 ; RRID: AB_11149861
Anti-mouse CD5, APC-eFluor 780	eBioscience	Cat# 47-0051-82; RRID: AB_2573940
Anti-mouse CD19, APC-eFluor 780	eBioscience	Cat# 47-0193-82; RRID: AB_10853189
Anti-mouse/human CD45R/B220, APC/Cyanine7	BioLegend	Cat# 103224; RRID: AB_313007
Anti-mouse CD16/32, APC/Cyanine7	BioLegend	Cat# 101328; RRID: AB_2104158
Anti-mouse FcεRIα, APC/Cyanine7	BioLegend	Cat# 134326; RRID: AB_2572064
Anti-human/mouse CD11b, APC/Cyanine7	Tonbo Biosciences	Cat# 25-0112-U100; RRID: AB_3094465
Anti-mouse CD11c, APC/Cyanine7	Tonbo Biosciences	Cat# 25-0114; RRID: AB_2621626
Anti-mouse Ly-6G, APC/Cyanine7	Tonbo Biosciences	Cat# 25-1276; RRID: AB_2621632
Anti-mouse TER-119, APC-eFluor 780	eBioscience	Cat# 47-5921-82; RRID: AB_1548786
Streptavidin, PE-Cyanine7	eBioscience	Cat# 25-4317-82; RRID: AB_10116480
Anti-mouse CD45.2, Alexa Fluor 700	eBioscience	Cat# 56-0454-82; RRID: AB_657752

Anti-mouse IL-13, Alexa Fluor 488	eBioscience	Cat# 53-7133-82; RRID: AB_2016708
Anti-mouse GATA3, BV421	BD Biosciences	Cat# 563349; RRID: AB_2738152
Anti-mouse/human IL-5, APC	BioLegend	Cat# 504306; RRID: AB_315330
Anti-mouse Amphiregulin biotin	R&D Systems	Cat# BAF989; RRID: AB_2060662
Anti-mouse CD16/CD32	eBioscience	Cat# 14-0161-85; RRID: AB_467134
Anti-mouse/human IL-5, PE	BD Biosciences	Cat# 554395; RRID: AB_395364
Anti-mouse CD45.1, PE-Cyanine7	eBioscience	Cat# 25-0453-82; RRID: AB_469629
Streptavidin, APC	BioLegend	Cat# 405207
Anti-mouse CD16/CD32	eBioscience	Cat# 14-0161-85; RRID: AB_467134
Anti-human CD303α, FITC	eBioscience	Cat# 11-9818-42; RRID: AB_11149122
Anti-human CD94, FITC	eBioscience	Cat# 11-0949-42; RRID: AB_11149673
Anti-human CD19, FITC	Tonbo Biosciences	Cat# 35-0198; RRID: AB_2621683
Anti-human CD123, FITC	eBioscience	Cat# 11-1239-42; RRID: AB_10854578
Anti-human CD3, FITC	eBioscience	Cat# 11-0036-42; RRID: AB_1272072

Anti-human CD16, FITC	eBioscience	Cat# 11-0168-42; RRID: AB_10805747
Anti-human CD14, FITC	Tonbo Biosciences	Cat# 35-0149; RRID: AB_2621680
Anti-human Amphiregulin biotin	R&D Systems	Cat# BAF262; RRID: AB_2060677
Anti-human CD45, Alexa Fluor700	BioLegend	Cat# 304024; RRID: AB_493761
Anti-human CD127 (IL-7Ra), Brilliant Violet 421	BioLegend	Cat# 351310; RRID: AB_10960140
Anti-mouse CD3, FITC	BioLegend	Cat# 100204; RRID: AB_312661
Anti-mouse CD5, FITC	eBioscience	Cat# 11-0051-82; RRID: AB_464908
Anti-mouse CD16/32, FITC	BioLegend	Cat# 101306; RRID: AB_312805
Anti-mouse/human CD11b, FITC	BioLegend	Cat# 101206; RRID: AB_312789
Anti-mouse CD11c, FITC	BioLegend	Cat# 117306; RRID: AB_313775
Anti-mouse CD19, FITC	Tonbo Biosciences	Cat# 35-0193; RRID: AB_2621682
Anti-mouse/human CD45R/B220, FITC	BioLegend	Cat# 103206; RRID: AB_312991
Anti-mouse TER-119/Erythroid Cells, FITC	BioLegend	Cat# 116206; RRID: AB_313707
Anti-mouse Ly-6G, FITC	Tonbo	Cat# 35-1276; RRID:

	Biosciences	AB_2621704
Anti-mouse FcεRIα, FITC	BioLegend	Cat# 134306; RRID: AB_1626108
Anti-mouse CD127 (IL-7Rα), PE	BioLegend	Cat# 135010; RRID: AB_1937251
Anti-mouse CD127 (IL-7Rα), APC	BioLegend	Cat# 135012; RRID: AB_1937216
Anti-mouse KLRG1, PerCP-eFluor 710	eBioscience	Cat# 46-5893-82; RRID: AB_10670282
Anti-mouse CD45.2, Pacific Blue	BioLegend	Cat# 109820; RRID: AB_492872
Anti-IL13 Rabbit Polyclonal antibody	Affinity	Cat# DF2520; RRID: AB_2839726
Anti-TMX2 Rabbit Polyclonal antibody	Proteintech	Cat# 19838-1-AP; RRID: AB_10642435
HRP-conjugated Goat Anti-Mouse IgG (H+L)	Proteintech	Cat# SA00001-1; RRID: AB_2722565
HRP-conjugated Affinipure Goat Anti-Rabbit IgG (H+L)	Proteintech	Cat# SA00001-2; RRID: AB_2722564
Anti-PERK (C33E10) Rabbit mAb	Cell Signaling Technology	Cat# 3192; RRID:AB_2095847
Anti-Phospho-PERK (Thr980) (16F8) Rabbit mAb	Cell Signaling Technology	Cat# 3179, RRID:AB_2095853
Anti-Phospho-PERK-T982 Rabbit pAb	ABclonal	Cat# AP0886; RRID:AB_2771413
Anti-eIF2α (D7D3) Rabbit mAb	Cell Signaling Technology	Cat# 5324; RRID:AB_10692650

Anti-Phospho-eIF2α (Ser51) (D9G8) Rabbit mAb	Cell Signaling Technology	Cat# 3398; RRID:AB_2096481
Anti-Xbp-1s (E9V3E) Rabbit mAb	Cell Signaling Technology	Cat# 40435; RRID: AB_2891025
Anti-ATF6 (70B1413.1) Mouse mAb	Novus	Cat# NBP1-40256, RRID:AB_2058774
Anti-β3-Tubulin (TU-20) Mouse mAb	Cell Signaling Technology	Cat# 4466, RRID:AB_1904176
Chemicals, Peptides, and Recombinant Proteins		
4μ8c	TOPSCIENCE	Cat# T6363
Ceapin-A7	TOPSCIENCE	Cat# T9110
ISRIB	TOPSCIENCE	Cat# T2027
Tunicamycin	TOPSCIENCE	Cat# T13229
GSK2606414	MedChemExpress	Cat# HY-18072
CCT020312	MedChemExpress	Cat# HY-119240
Deoxycholic acid	Sigma	Cat# D2510
Deoxycholic acid-iFluor 647 conjugate	AAT Bioquest	Cat# 36705
Sodium dodecyl sulfate	Beyotime	Cat# ST2681
Recombinant Human TMX2 (N-6His)	Novoprotein	Cat# CH30
Recombinant Human Insulin	Solarbio	Cat# I8830
RIPA lysis buffer	Servicebio	Cat# G2002
Protein Phosphatase Inhibitor (All-in-one, 100×)	Solarbio	Cat# P1260
Enhanced luminescent liquid	Biosharp	Cat# BL520B
Hybridization Nitrocellulose Filter	Merck Millipore	Cat# HATF00010
Protease Inhibitor Cocktail (EDTA-Free,	MedChemExpress	Cat# HY-K0010

100× in DMSO)		
PMSF (100 mM)	Solarbio	Cat# P0100
Penicillin-Streptomycin, 100×	MACGENE	Cat# CC004
Recombinant Murine IL-2	Peprotech	Cat# 212-12
Recombinant Mouse IL-7 (carrier-free)	Biolegend	Cat# 577802
Recombinant Murine IL-17E	Peprotech	Cat# 210-17E
Recombinant Murine IL-33	Peprotech	Cat# 210-33
Dextran Sulfate Sodium Salt	MP Biomedicals	Cat# 160110
Phorbol 12-myristate 13-acetate (PMA)	BioGems	Cat# 1652981
Ionomycin calcium salt	BioGems	Cat# 5608212
Brefeldin A	BioGems	Cat# 2031560
DNase I	Sigma	Cat# DN25
Collagenase VIII	Sigma	Cat# C2139
HEPES	Solarbio	Cat# H8090
EDTA	Solarbio	Cat# E1170
Phosphate Buffer Saline	Solarbio	Cat# P1010
Dimethyl sulfoxide	Solarbio	Cat# D8317
Isopropanol	FUYU CHEMICAL	CAS# 67-63-0
Ethanol	FUYU CHEMICAL	CAS# 64-17-5
Fetal Bovine Serum	GIBCO	Cat# 10099141C
RPMI 1640	MACGENE	Cat# CM10041
Vancomycin hydrochloride	MACKLIN	Cat# V820413
Fixation/Permea-bilizationConcentrate	eBioscience	Cat# 00-5223-56
IMDM	MACGENE	Cat# CM10016
Methanol	Fisher Scientific	Cat# DN25

RNA isolater Total RNA Extraction Reagent	Vazyme	Cat# R401-01
Percoll	Cytiva	Cat# 17089109
Biological samples		
Human: feces of patients with UC	Qilu hospital of Shandong University	See Table S2
Critical commercial assays		
SMART-Seq HT Kit	TaKaRa	Cat# 634437
Nextera XT Library Prep Kit	Illumina	Cat# 15032350
Nextera XT Index Kit	Illumina	Cat# 15052163
AMPure XP Beads	Beckman Coulter	Cat# A63881
TruePrep Index Kit V2 for Illumina	Vazyme	Cat# TD202
Qubit ssDNA Assay Kit	Thermo Fisher Scientific	Cat# Q10212
Zombie Aqua™ Fixable Viability Kit	BioLegend	Cat# 423102
Hoechst Staining Kit	Beyotime	Cat# C0003
Mito-Tracker Green	Beyotime	Cat# C1048
ER-Tracker Green	Beyotime	Cat# C1042S
BCA Protein Assay Kit	Beyotime	Cat# P0011
Multiplex Assay Kit for IL5, IL13, AREG, etc. by FLIA (Flow Luminescence Immunoassay)	Cloud-Clone Corp.	Cat# IS135-Mouse
TNT® T7 Quick Coupled Transcription/Translation System	Promega	Cat# L1170
PROTEOSTAT® Aggresome detection kit	Enzo	Cat# ENZ-51035
Total Bile Acid (TBA) Colorimetric Assay	Elabscience	Cat# E-BC-K181-M

Kit		
Experimental Models: Organisms/Strains		
Mouse: C57BL/6	Gempharmatech	Cat# N000013
Mouse: *IL5*$^{RFP\text{-}Cre}$	Jackson Laboratories	Cat# R5/+
Mouse: Rag2$^{-/-}$Il2rg$^{-/-}$	Taconics Biosciences	Cat# 4111
Mouse: Nr1h4$^{-/-}$	Gempharmatech	Cat# T012640
Mouse: Gpbar1$^{-/-}$	Gempharmatech	Cat# T012754
Deposited Data		
RNA-seq data; ILC2s treated with DCA	This paper	GEO: GSE294014
RNA-seq data; ILC2s treated with FK866	Shen J et al. 2022	GEO: GSE218368
Software and Algorithms		
Progenesis QI	Waters Corporation	N/A
Biacore T200 Evaluation Software	Cytiva	N/A
ImageJ 1.52v	NIH	https://fiji.sc/
FlowJo version 10.4.2	FlowJo	https://www.flowjo.com
Prism 8.0.1	GraphPad Software	https://www.graphpad.com
Other		
Normal control diet	Jiangsu Xietong Pharmaceutical Bio-engineering Co., Ltd	Cat# XT93G
BA-containing diets	This paper; Jiangsu Xietong	N/A
