# Supplementary material for: Vancomycin eliminates gut deoxycholic acid, restoring ER proteostasis in ILC2s and relieving colitis
Source: JCI Insight. 2026 Apr 8;11(7):e197470. doi: 10.1172/jci.insight.197470 (PMC13134711; doi:10.1172/jci.insight.197470)

Full unedited gel for Figure 4H

anti-Areg

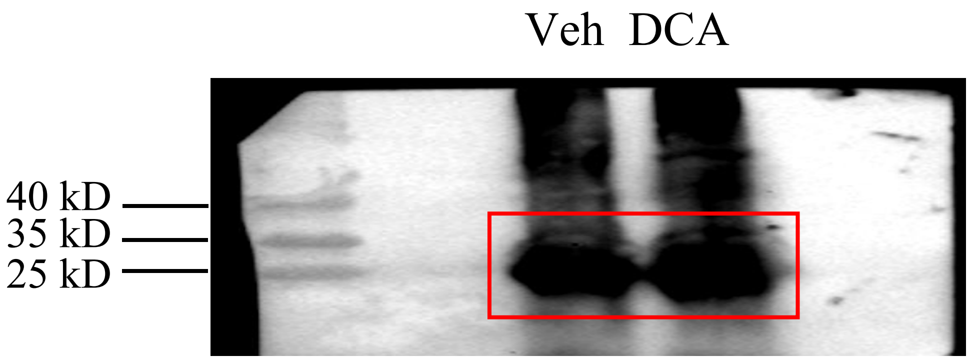

Full unedited gel for Figure 4H

anti-IL-5

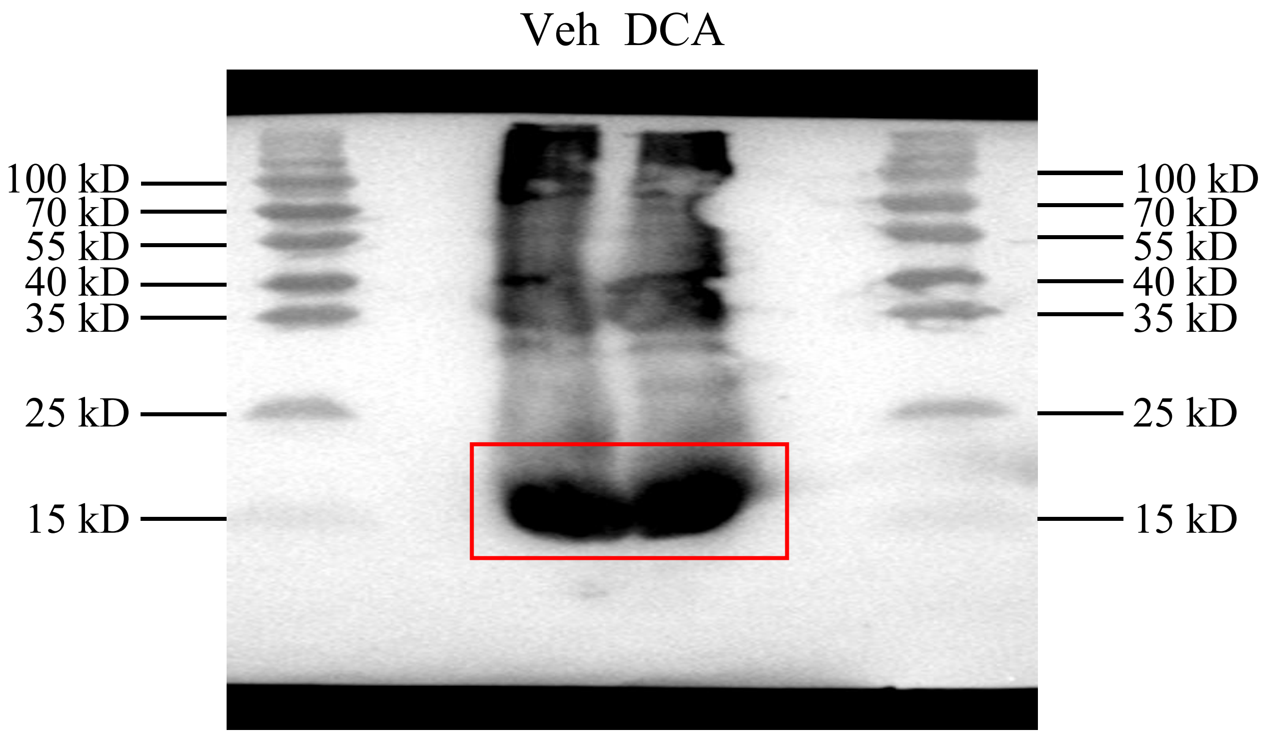

Full unedited gel for Figure 4H

anti-IL-13

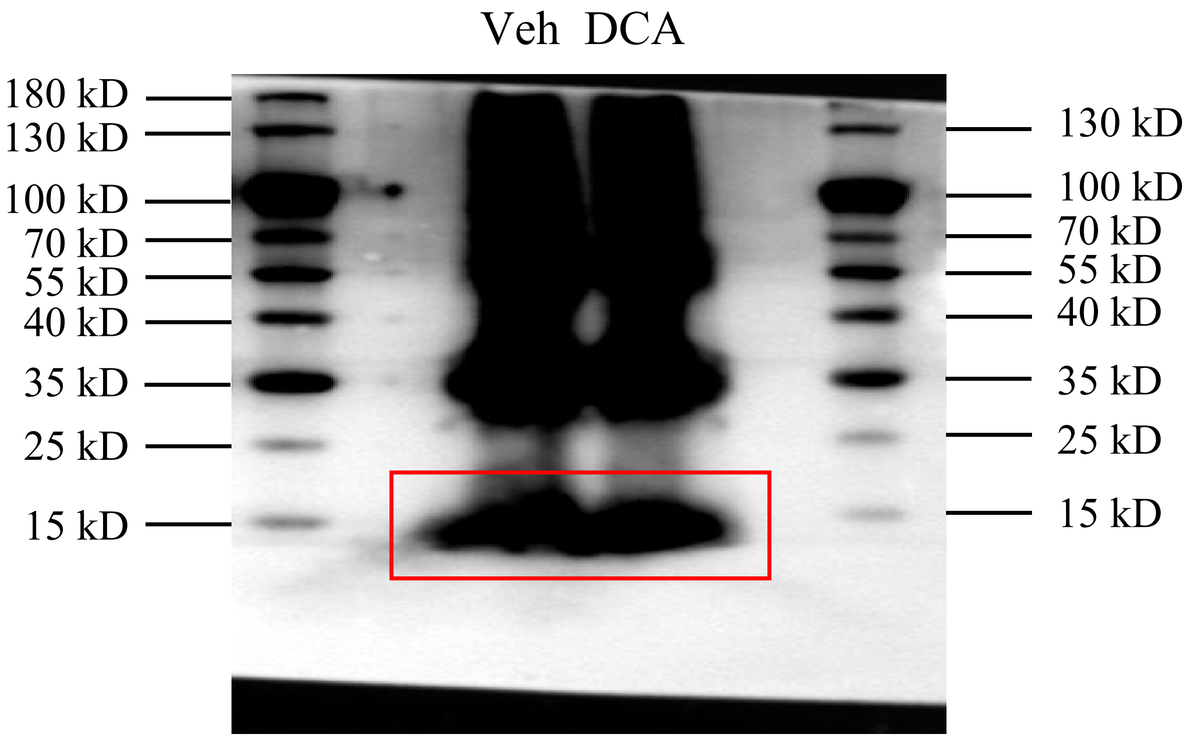

Full unedited gel for Figure 5E

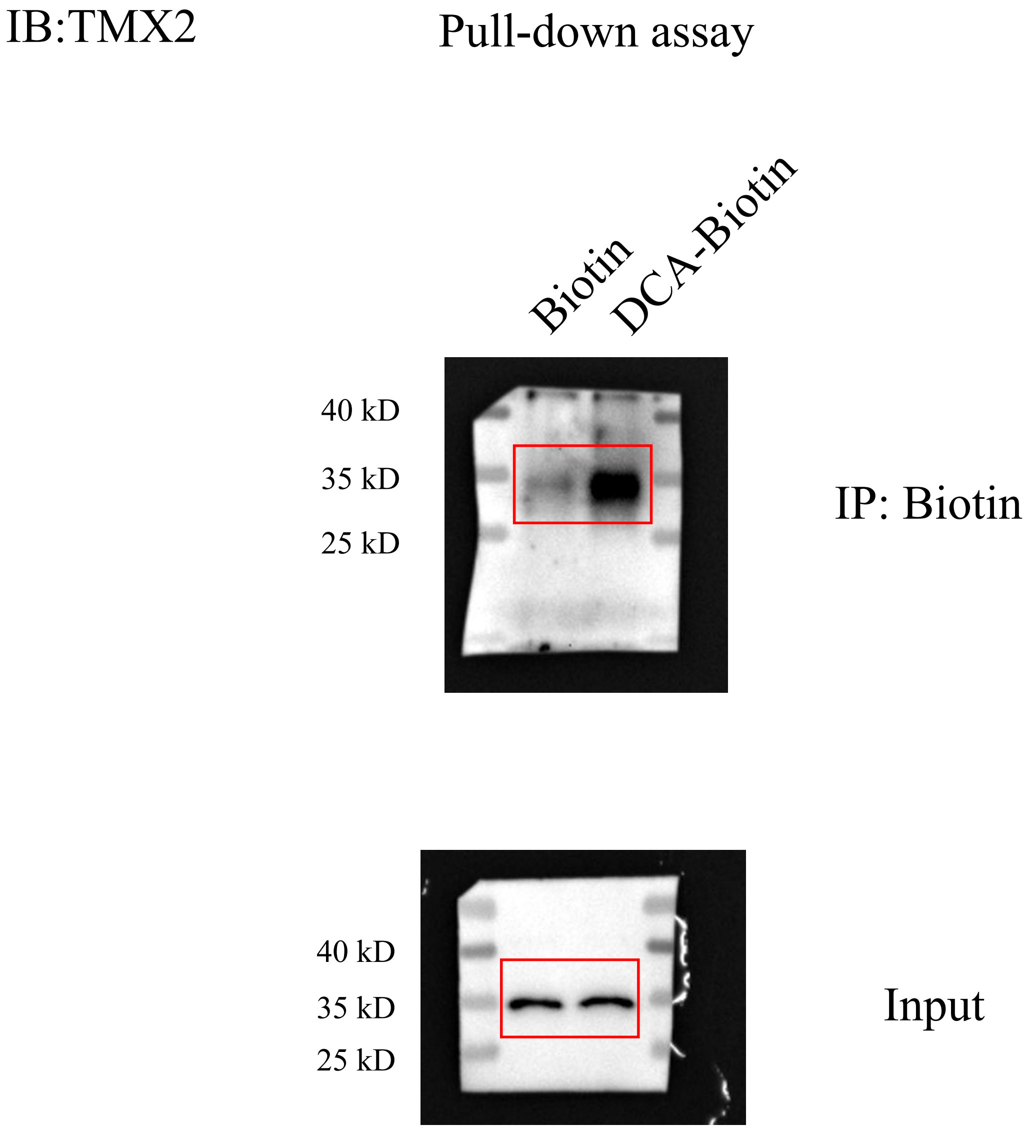

Full unedited gel for Figure 6B

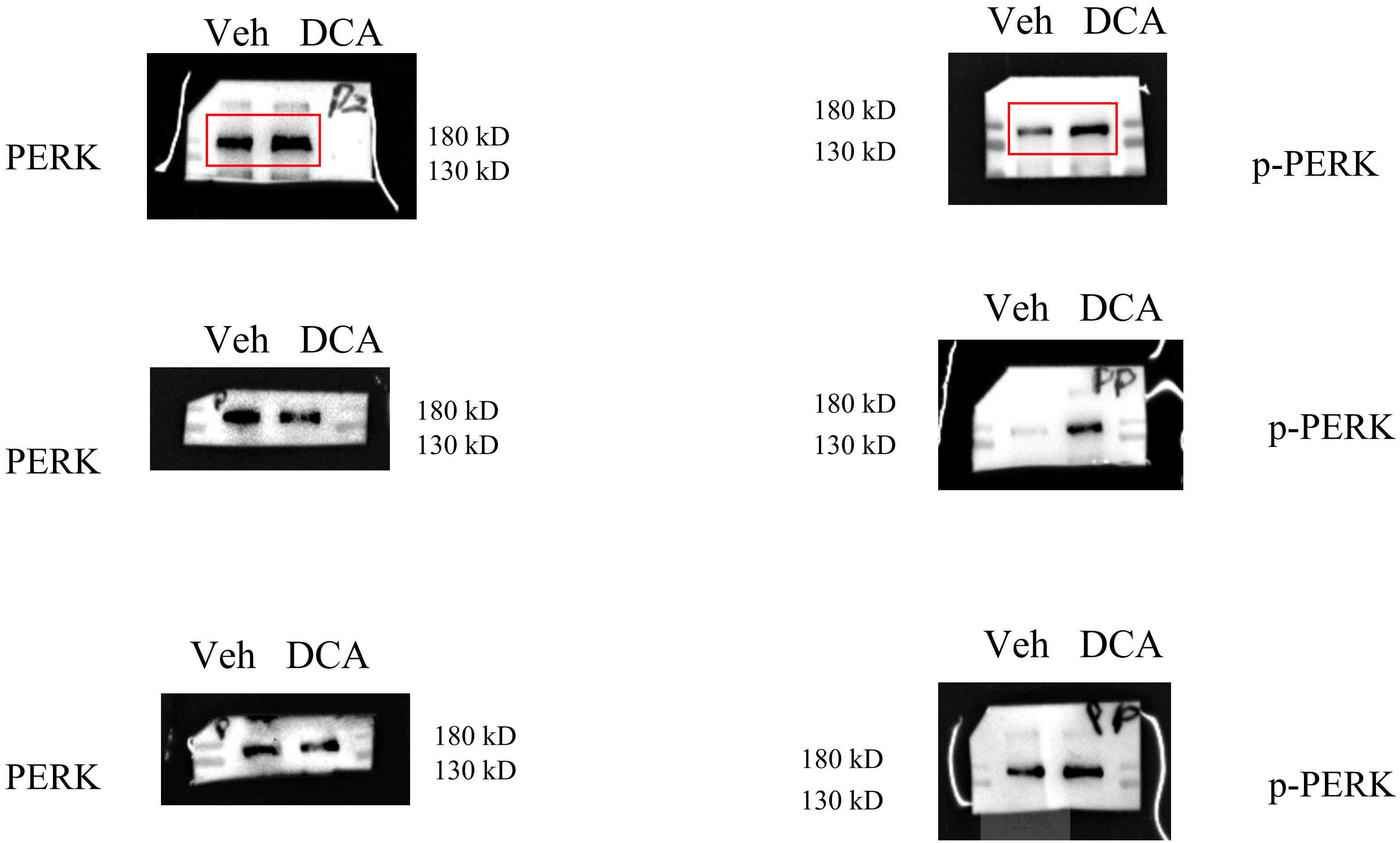

Full unedited gel for Figure 6B

Veh DCA

eIF2α

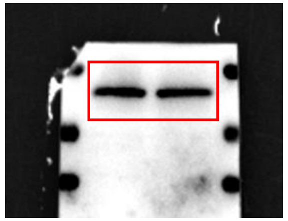

40 kD
35 kD
25 kD

Veh DCA

40 kD
35 kD

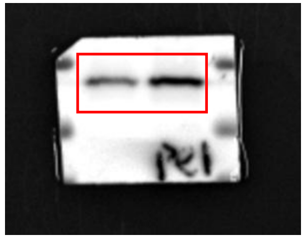

p-eIF2α

Veh DCA

eIF2α

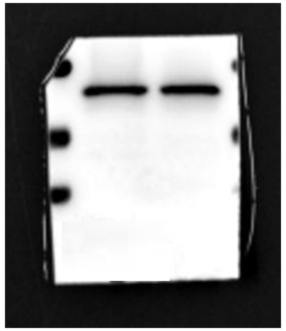

40 kD
35 kD
25 kD

Veh DCA

40 kD
35 kD

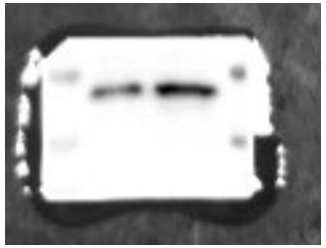

p-eIF2α

Veh DCA

eIF2α

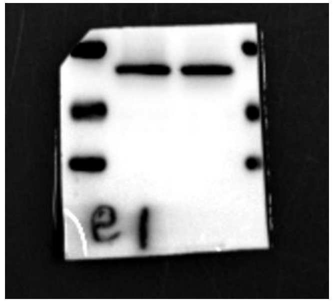

40 kD
35 kD
25 kD

Veh DCA

40 kD
35 kD
25 kD

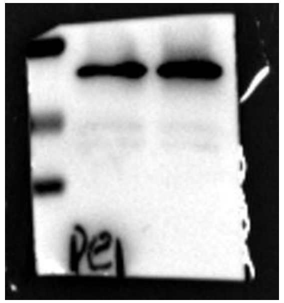

p-eIF2α

Full unedited gel for Figure 6B

Veh DCA

ATF6

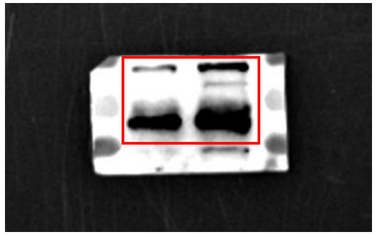

100 kD
70 kD
55 kD

Veh DCA

ATF6

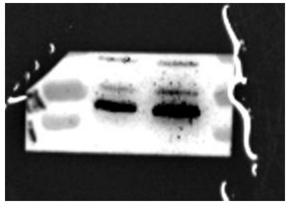

100 kD
70 kD
55 kD

Veh DCA

ATF6

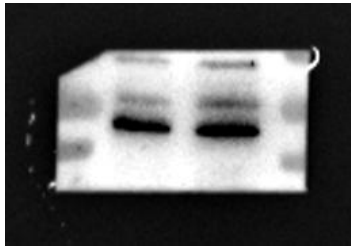

100 kD
70 kD
55 kD

Veh DCA

70 kD
55 kD

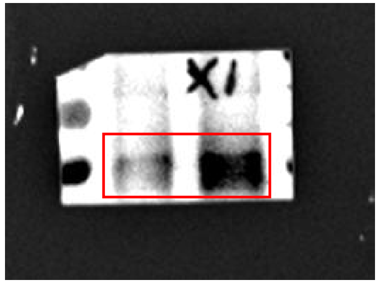

Xbp1s

Veh DCA

70 kD
55 kD

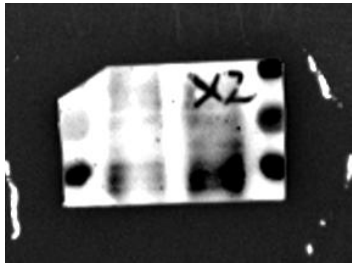

Xbp1s

Veh DCA

70 kD
55 kD

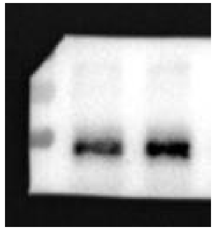

Xbp1s

Full unedited gel for Figure 6B

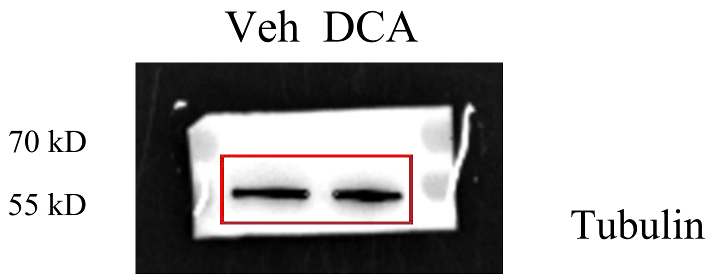

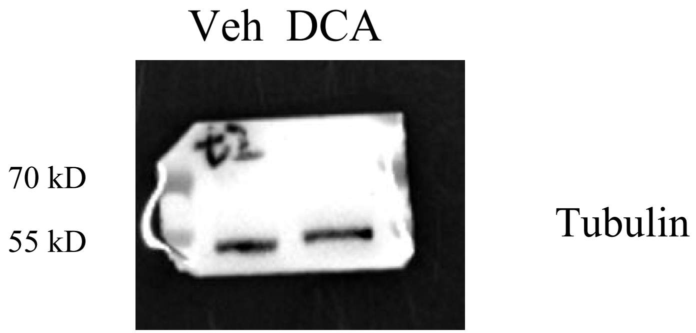

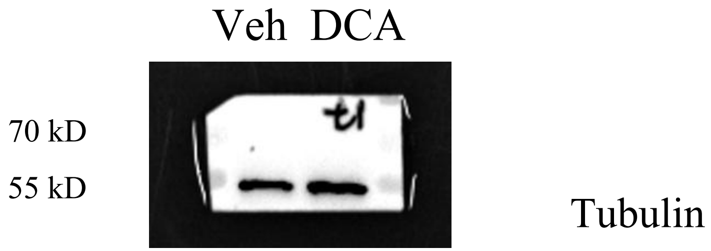

Supplement: Unedited blot and gel images [file jciinsight-11-197470-s222.pdf]
